# Brain transcriptomes of zebrafish and mouse Alzheimer's disease knock-in models imply early disrupted energy metabolism

**DOI:** 10.1242/dmm.049187

**Published:** 2022-01-26

**Authors:** Karissa Barthelson, Morgan Newman, Michael Lardelli

**Affiliations:** Alzheimer's Disease Genetics Laboratory, School of Biological Sciences, University of Adelaide, North Terrace, Adelaide, SA 5005, Australia

**Keywords:** Alzheimer's disease, Zebrafish, Mouse, RNA-seq, Oxidative phosphorylation, Brain

## Abstract

Energy production is the most fundamentally important cellular activity supporting all other functions, particularly in highly active organs, such as brains. Here, we summarise transcriptome analyses of young adult (pre-disease) brains from a collection of 11 early-onset familial Alzheimer's disease (EOFAD)-like and non-EOFAD-like mutations in three zebrafish genes. The one cellular activity consistently predicted as affected by only the EOFAD-like mutations is oxidative phosphorylation, which produces most of the energy of the brain. All the mutations were predicted to affect protein synthesis. We extended our analysis to knock-in mouse models of *APOE* alleles and found the same effect for the late onset Alzheimer's disease risk allele ε4. Our results support a common molecular basis for the initiation of the pathological processes leading to both early and late onset forms of Alzheimer's disease, and illustrate the utility of zebrafish and knock-in single EOFAD mutation models for understanding the causes of this disease.

## Abstract (precis)

**Summary:** Young adult zebrafish mutants and a mouse model of a genetic variant promoting early- and late-onset Alzheimer's disease, respectively, share changes in brain gene expression, indicating disturbance of oxidative phosphorylation.

## INTRODUCTION

Alzheimer's disease (AD) is a complex and highly heterogenous neurodegenerative disease, defined by the presence of intracellular neurofibrillary tangles (primarily consisting of hyperphosphorylated tau proteins), and extracellular plaques mostly consisting of a small peptide, amyloid β (Aβ) (Jack et al., 2018). The pathological basis of AD has been the subject of research for over 100 years (Alzheimer, 1906). Nevertheless, most treatments tested in clinical trials have shown limited therapeutic benefit.

AD has a strong genetic basis (reviewed by Sims et al., 2020). In some rare cases, early-onset familial forms of AD (EOFAD, occurring before 65 years of age) arise due to dominant mutations in one of four genes: presenilin 1 (*PSEN1*), presenilin 2 (*PSEN2*), amyloid β precursor protein (*APP*) and sortilin-related receptor 1 (*SORL1*) (reviewed by Barthelson et al., 2020a; Bertram and Tanzi, 2012; Ayodele et al., 2021). However, most AD cases are sporadic, showing symptom onset after the arbitrarily defined threshold of 65 years (late-onset sporadic AD, LOAD). Genetic variants at many loci have been associated with increased risk of LOAD (Jansen et al., 2019; Kunkle et al., 2019; Lambert et al., 2013). The most potent variant is the ε_4_ allele of apolipoprotein e (*APOE*) (Farrer et al., 1997), which has been described as ‘semi-dominant’ (Genin et al., 2011).

An understanding of the early cellular stresses on the brain that eventually lead to AD is necessary to advance the development of preventative treatments. This is difficult to achieve through studying living humans, as EOFAD mutations are rare, and access to young presymptomatic brains is limited. Nevertheless, imaging studies have implicated structural and functional changes to the brain long before diagnosis of AD (Iturria-Medina et al., 2016; Quiroz et al., 2015). Brain imaging cannot provide detailed molecular information about these changes. Transcriptome analysis is, currently, the strategy that can provide the highest resolution molecular description of cells and tissues. However, transcriptome analyses of ante-mortem brains carrying EOFAD mutations can only be performed using brain tissue from animal models.

Our group has exploited the zebrafish to generate a collection of knock-in models of EOFAD-like mutations in order to analyse their young brain transcriptomes (Barthelson et al., 2020b, 2021a, 2021b, 2021c; Dong et al., 2021; Hin et al., 2020a, 2021; Jiang et al., 2020; Newman et al., 2019). Our experimental philosophy has been to replicate, as closely as possible, the single heterozygous mutation state of EOFAD in humans, thereby avoiding possibly misleading assumptions regarding the molecular mechanism(s) underlying the disease. Our overall goal has been to compare a broad range of EOFAD-like mutations in a number of EOFAD genes to define their shared pathological effects in young adult brains where the long progression to AD begins. To assist in this definition (by exclusion), we also created non-EOFAD-like mutations in the same genes as negative controls, i.e. frameshift mutations in the presenilin genes that do not cause EOFAD (reviewed by Jayne et al., 2016, the ‘reading frame preservation rule’). The presentation of EOFAD and LOAD as similar diseases (reviewed by Blennow et al., 2006; Masters et al., 2015) implies similarity, to some degree, at the cellular and molecular levels. Therefore, despite differences in the genetic variants that promote these two diseases, understanding the molecular effects of heterozygosity for EOFAD mutations may give insight into the molecular changes underpinning LOAD.

Here, we summarise our findings of brain transcriptome analyses of EOFAD-like mutations in the zebrafish orthologues of genes implicated in EOFAD: *psen1*, *psen2* and *sorl1*. EOFAD mutations also exist in *APP*. However, zebrafish express two *APP* ‘co-orthologous’ genes, *appa* and *appb*, complicating analysis of single heterozygous mutations. Therefore, we re-analysed the best available publicly accessible brain transcriptomic data from a knock-in model of *APP* mutations: the *App^NL-G-F^* mouse. Finally, we compared whether the brain transcriptome changes occurring due to single heterozygous EOFAD-like mutations in zebrafish are similar to the changes occurring due to the strongest genetic risk factor for LOAD, the ε_4_ allele of *APOE*, using publicly available brain transcriptome data from a humanised *APOE* targeted-replacement mouse model (APOE-TR) (Sullivan et al., 1997). We identify changes to energy metabolism as the earliest detectable cellular stress due to AD mutations, and demonstrate that knock-in zebrafish models are valuable tools for studying the earliest molecular pathological events in this disease.

## RESULTS

### Transcriptome analysis of zebrafish models of EOFAD

**Fig. 1.**
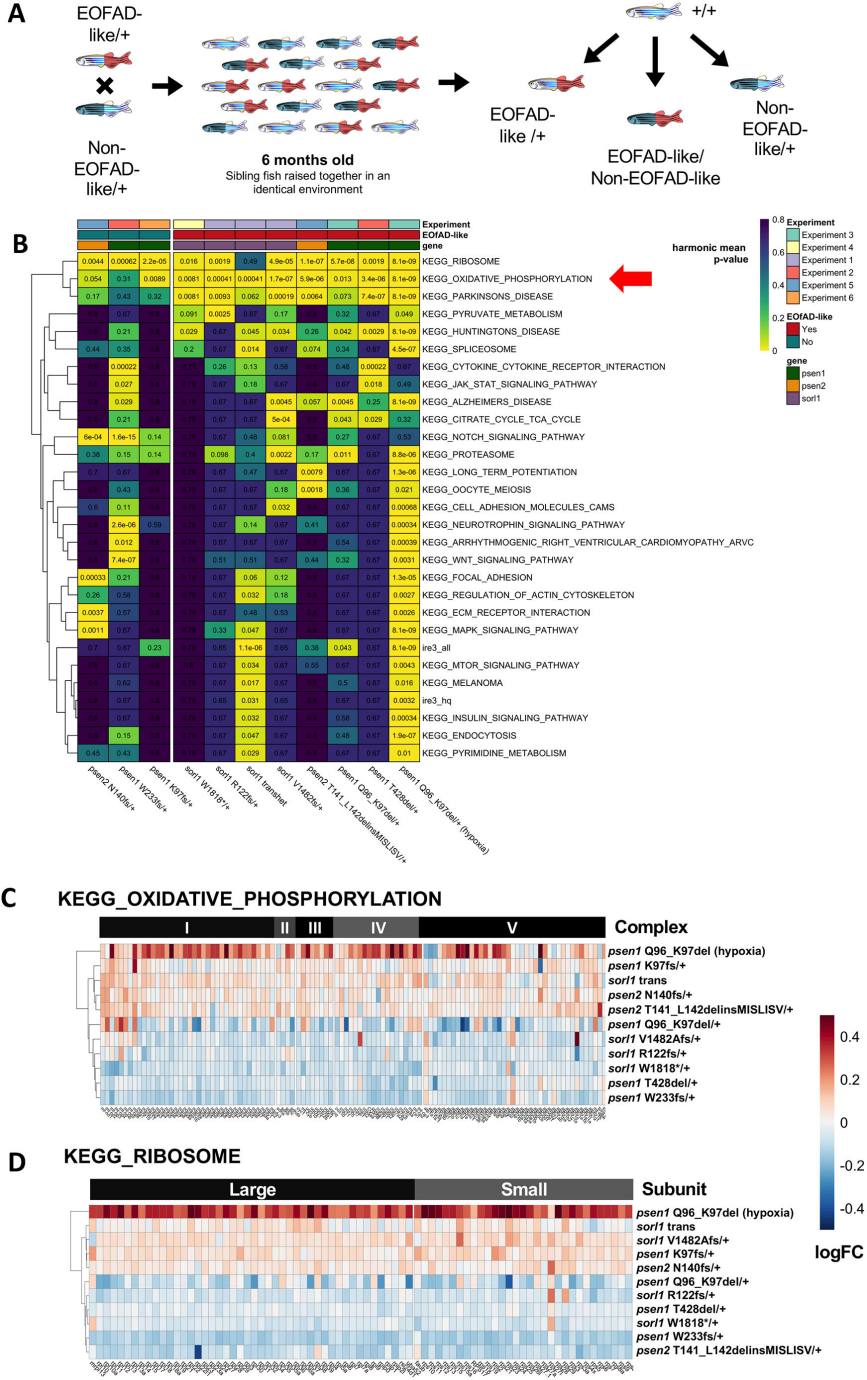
**RNA-seq analysis of 6-month-old zebrafish models of EOFAD.** (A) Schematic of an RNA-seq experiment using zebrafish. A single mating of a single pair of fish heterozygous for either an EOFAD-like or a non-EOFAD-like mutation results in a family heterozygous mutant, transheterozygous mutant and wild-type siblings. Comparisons made between genotypes in an RNA-seq experiment are depicted. (B) Heatmap summary of significantly altered KEGG and IRE gene sets in zebrafish EOFAD genetic models at 6 months of age. Only gene sets significantly altered (FDR-adjusted harmonic mean *P*<0.05) in at least two comparisons of mutant zebrafish to their corresponding wild-type siblings are shown. Columns are grouped by whether or not the zebrafish genotype is EOFAD-like, and rows are clustered based on their Euclidean distance. The numbers are FDR-adjusted harmonic mean *P*-values. (C,D) Heatmap indicating the log fold change (logFC) of genes in the KEGG gene sets for oxidative phosphorylation (C) and the ribosome (D) in zebrafish mutants compared to their wild-type siblings. Rows are clustered based on their Euclidean distance, and columns are grouped by the complex in the electron transport chain to which an encoded protein belongs (C), or whether an encoded protein forms part of the large or small ribosomal subunits (D). Only genes considered detectable in all RNA-seq experiments are depicted. See Figs S1-S6 and Table 1 for more information on individual study designs.

We first collated our findings from our zebrafish models of EOFAD-like mutations in *psen1* (Barthelson et al., 2021a; Hin et al., 2020, 2021; Newman et al., 2019), *psen2* (Barthelson et al., 2021b) and *sorl1* (Barthelson et al., 2020b, 2021c)*.* An advantage of using zebrafish for RNA-seq analyses is minimisation of genetic and environmental noise through breeding strategies, such as that shown in Fig. 1A. Large families of synchronous siblings can consist of heterozygous mutant and wild-type genotypes, allowing direct comparisons of the effects of each mutation. So far, we have performed six brain transcriptomic analyses based on various breeding strategies (summarised in Table 1 and Figs S1-S6). The detailed analyses can be found in the publications cited above. However, the outcomes are summarised below and in Fig. 1.

**Table 1.**
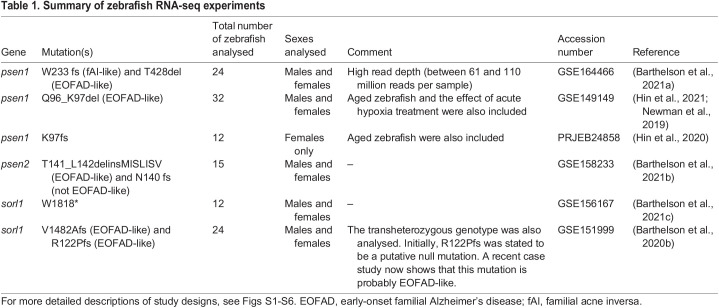
Summary of zebrafish RNA-seq experiments

In our previously published analyses, we found that heterozygosity for most of our EOFAD-like mutations does not result in many differentially expressed genes in young adult brains (as would be expected for modelling a disease that becomes overt in middle age) (Barthelson et al., 2020b, 2021a, 2021b, 2021c; Newman et al., 2019). Therefore, we performed gene set enrichment analyses (GSEA) to predict which cellular processes were affected by each of the mutations in each experiment. We used the Kyoto Encyclopedia of Genes and Genomes (KEGG) (Kanehisa and Goto, 2000) gene sets to determine whether changes to gene expression were observed in any of 186 biological pathways/processes. Additionally, we recently proposed that neuronal iron dyshomeostasis may be an effect-in-common of EOFAD mutations in the context of AD pathogenesis (Lumsden et al., 2018). Therefore, we used our recently defined iron responsive element (IRE) gene sets (Hin et al., 2021) to test for evidence of iron dyshomeostasis. Biological processes found to be affected in at least two different zebrafish mutants are shown in Fig. 1B (the statistical significance of all KEGG and IRE gene sets in each mutant can be found in Table S2). The one gene set consistently altered by all of the EOFAD-like mutations, but not by the non-EOFAD-like mutations examined, is the *KEGG_OXIDATIVE_PHOSPHORYLATION* gene set (Fig. 1C), supporting that changes to mitochondrial function are an early cellular stress in EOFAD. The *KEGG_OXIDATIVE_PHOSPHORYLATION* gene set is also affected by heterozygosity for the K97fs mutation of *psen1*. K97fs is a frameshift mutation and so does not follow the ‘reading frame preservation rule’ (Jayne et al., 2016) of presenilin EOFAD mutations. However, the truncated protein encoded by K97fs resembles a hypoxia-induced isoform of human *PSEN2*, denoted PS2V, which shows increased expression in LOAD brains (Sato et al., 1999, and see Moussavi Nik et al., 2015, for additional explanation). Therefore, K97fs is still an AD-relevant mutation.

Genes encoding the components of ribosomal subunits, as defined by the gene set *KEGG_RIBOSOME*, were affected by all the EOFAD-like mutations but also by non-EOFAD-like mutations in *psen1* and *psen2* (Fig. 1D). Evidence for iron dyshomeostasis was also observed for the relatively severe EOFAD-like mutation *psen1*^Q96_K97del/+^ (under both normoxia and acute hypoxia conditions) and in transheterozygous *sorl1* mutants (i.e. with complete loss of wild type *sorl1*), as shown by significant changes to the expression of genes possessing IRE(s) in the 3′ untranslated regions (UTRs) of their encoded mRNAs (*ire_hq* and *ire_all*).

### Transcriptome analysis of the APP^NL-G-F^ mouse model

EOFAD is also caused by mutations of the gene *APP.* Modelling of *APP* mutations in zebrafish is complicated by duplication of the *APP*-orthologous gene in this organism. However, brain transcriptome data are available for a knock-in mouse model of EOFAD mutations in *APP*: the *App^NL-G-F^* mouse model (Castillo et al., 2017). In this model, the murine *App* sequence is modified to carry humanised DNA sequences in the Aβ region, as well as the Swedish, Beyreuther/Iberian and Arctic EOFAD mutations (Saito et al., 2014). Although these mice do not closely reflect the genetic state of heterozygous human carriers of EOFAD mutations of *APP* (as the mice possess a total of six mutations within their modified *App* allele and are usually analysed as homozygotes), they should, at least, not generate artefactual patterns of gene expression change due to overexpression of transgenes (Saito et al., 2016). Castillo et al. (2017) performed brain transcriptomic profiling via microarrays of the brain cortices of male homozygous *App^NL-G-F^* mice relative to wild-type mice at 12 months of age, as well as a transgenic mouse model of AD, 3xTg-AD mice (Oddo et al., 2003) relative to non-Tg mice (Castillo et al., 2017). All mice used in the study were maintained as inbred lines. However, there is no information on whether any of the mice analysed were littermates. It is highly unlikely that the mice used in each comparison between mutant individuals and their wild-type counterparts all arose from the same litter, because obtaining three homozygous and three wild-type male mice in a single litter arising from an incrossing of heterozygous mutant mice (expected to produce a wild type:heterozygote:homozygote Mendelian genotype ratio of 1:2:1) would be a rare event, as litters of mice generally consist of five to ten pups. Therefore, additional variation was introduced into the analysis through the use of mice from different litters and this is likely confounding with genotype. This is important to note, as the results presented here were generated under the assumption that any effects of litter-of-origin are negligible. Our re-analysis of the microarray dataset of Castillo et al. (2017) aimed to address the following questions: (1) are the KEGG gene sets affected in the male homozygous *App^NL-G-F^* mice similar to those affected in EOFAD-like zebrafish?; and (2) is there evidence for iron dyshomeostasis in the brains of these mice? Initially, we attempted to replicate the results of Castillo et al. (2017) using the Affymetrix Transcriptome Analysis Console software. However, we were unable to find sufficient information to achieve this. Therefore, we analysed the microarray dataset in a reproducible manner following a recommended microarray analysis workflow (Carvalho and Irizarry, 2010).

**Fig. 2.**
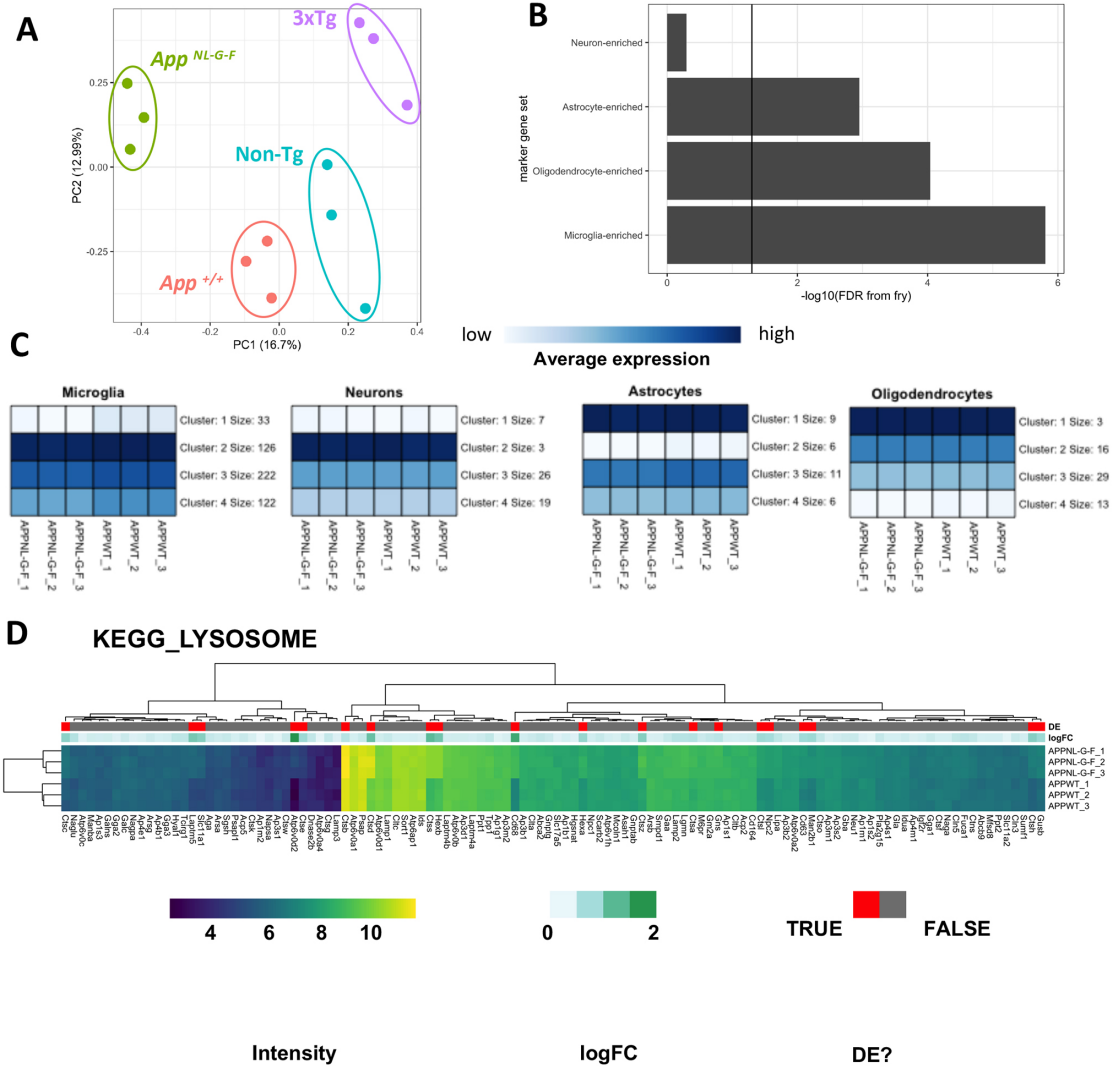
**Microarray analysis of male 12-month-old homozygous *App^NL-G-F^* mice.** (A) Principal component analysis of brain transcriptome data from male 12-month-old homozygous *App^NL-G-F^* (*n*=3), 3xTg (*n*=3), *App* wild-type (*App*^+/+^, *n*=3) and non-transgenic (non-Tg, *n*=3) mice. The numbers in parentheses indicate the percentage of variation in the dataset explained by a principal component. Each point represents a sample, which are coloured by genotype. (B) Bar chart showing the FDR-adjusted *P*-value (directional hypothesis) from fry on marker genes of neurons, oligodendrocytes, astrocytes and microglia in *App^NL-G-F^* relative to wild type. (C**)** Heatmaps indicating the expression (intensity) of genes within these marker gene sets summarised using K-means (K=4). (D) Heatmap showing the expression of genes in the *KEGG_LYSOSOME* gene set, clustered by their Euclidean distance. Each gene is labelled in red if they were identified as differentially expressed (DE), and the magnitude of the fold change (logFC) is shown in green.

After pre-processing of the raw intensities (Fig. S7), we performed principal component analysis (PCA) to explore the overall similarity between samples (Fig. 2A). Samples separated across PC1 by genotype, suggesting that the homozygous genotypes in this study result in distinct transcriptome states. Notably, the *App^NL-G-F/NL-G-F^* samples and their corresponding *App^+/+^* control samples appeared to separate to a greater extent across PC1 than the 3xTg samples and their corresponding non-Tg wild-type control samples. Additionally, a differential gene expression analysis revealed 158 and 126 genes to be differentially expressed in *App^NL-G-F/NL-G-F^* and 3xTg mice, respectively (relative to their corresponding controls, Fig. S8). This suggests that the disturbance to the cortex transcriptome in *App^NL-G-F/NL-G-F^* mice is greater than in 3xTg mice.

We did not observe alteration of any similar gene sets between the *App^NL-G-F^* mice and our EOFAD-like zebrafish (Figs S9-S12). However, any similarities may well have been masked by the overwhelming effects of greater age, variable environment (mouse litter-of-origin) and the effects of their six *App* mutations on brain cortex cell-type proportions and inflammatory processes. The most statistically significantly affected cellular process in 12-month-old *App^NL-G-F^* mice was lysosomal function, as represented by the *KEGG_LYSOSOME* gene set (Fig. 2D). Additionally, a plethora of inflammatory gene sets were also affected, with changes in the relative proportions of glial cells, particularly microglia, contributing to the appearance of increased levels of these gene transcripts in the bulk cortex RNA analysed (Fig. 2B,C; Fig. S13). Note that changes to cell-type proportions are not observed in our zebrafish models of EOFAD (Barthelson et al., 2020b, 2021b, 2021c) (see Fig. S14 for two examples).

### Do changes to gene expression in the oxidative phosphorylation pathway also occur in LOAD?

A puzzling observation from the genome-wide association studies (GWAS) of LOAD, is that none of the risk variants identified fall within the EOFAD genes *PSEN1*, *PSEN2* or *APP* (Jansen et al., 2019; Kunkle et al., 2019; Lambert et al., 2013). This has led to speculation that EOFAD and LOAD may be distinct diseases despite their histopathological and cognitive similarities (reviewed by DeTure and Dickson, 2019; Tellechea et al., 2018). Only one gene identified by GWAS of LOAD, *SORL1*, is suspected to harbour mutations causative of EOFAD. Mutations in *SORL1* cause AD with ages of onset typically later than many mutations in *PSEN1* or *APP* (or maybe incompletely penetrant) (Pottier et al., 2012; Thonberg et al., 2017). Nevertheless, as shown in Fig. 1B, we identified changes in the KEGG gene set for oxidative phosphorylation in young adult zebrafish heterozygous for EOFAD-like mutations in *sorl1*, as well as in zebrafish modelling overexpression of the PS2V isoform that is upregulated in LOAD (K97 fs).

**Fig. 3.**
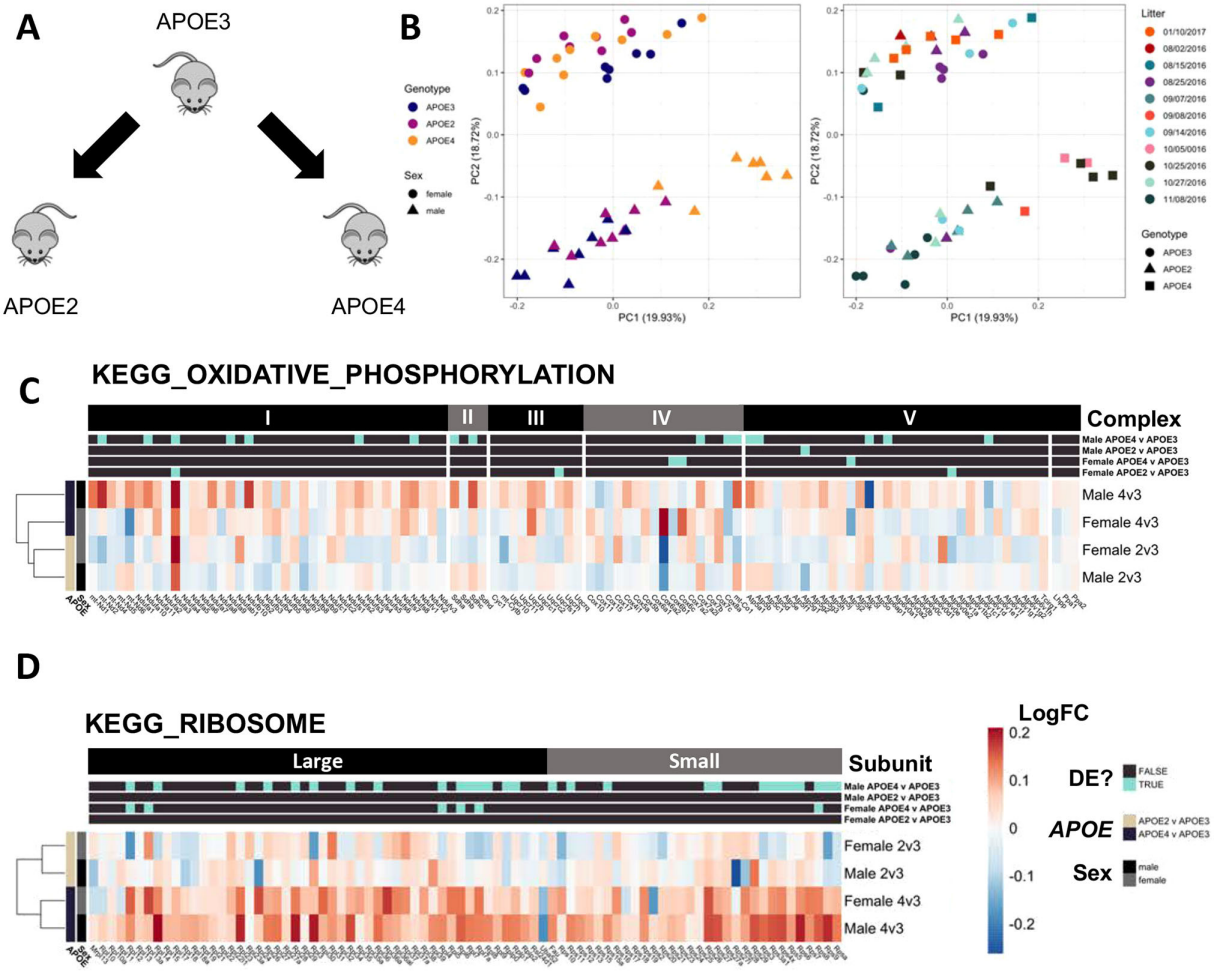
**RNA-seq analysis of 3-month-old APOE-TR mice.** (A) Visual representation of the comparison of APOE4 (*n*=7 males and 9 females) or APOE2 (*n*=8 males and 8 females) mice to APOE3 (*n*=8 males and 8 females). This comparison was made for both male and female mice separately. (B) PCA of 3-month-old APOE-TR mice. Principal component 1 (PC1) is plotted against PC2. The numbers in parentheses indicate the percentage of variation in the dataset explained by a principal component. In the left graph, each point represents a sample, which are coloured by *APOE* genotype and shaped by sex. In the right plot, each point is coloured according to litter (implied from the date of birth of each mouse), and shaped by *APOE* genotype. (C,D**)** Heatmap showing the log fold change (logFC) of genes in the *KEGG_OXIDATIVE_PHOSPHORYLATION* (C) and *KEGG_RIBOSOME* (D) gene sets in APOE-TR mice. Rows are clustered based on their Euclidean distance, and columns are grouped by the complex in the electron transport chain to which an encoded protein belongs (C), or whether an encoded protein forms part of the large or small ribosomal subunits (D). Genes are labelled in blue above if they were classified as differentially expressed (DE, FDR<0.05) in the differential gene expression analysis in the listed comparisons.

The strongest and most common genetic risk factor for LOAD is the ε_4_ allele of the gene *APOE* (Corder et al., 1993; Genin et al., 2011; Jansen et al., 2019; Kunkle et al., 2019; Lambert et al., 2013; Saunders et al., 1993). Like *APP*, the *APOE* orthologous gene in zebrafish is refractory to analysis due to duplication. Therefore, to compare our zebrafish mutant data to early brain transcriptome changes caused by the ε_4_ allele of *APOE*, we analysed data from a set of human gene-targeted replacement mouse models, APOE-TR (Sullivan et al., 1997). These mouse models transcribe human *APOE* alleles from the endogenous murine *Apoe* promotor: the predominant human allele ε_3_; the rare AD-protective ε_2_ allele; and the AD-risk allele ε_4_. Zhao et al. (2020) performed a comprehensive brain transcriptome profiling experiment across aging in both male and female mice to assess the effect of homozygosity for the ε_2_ or ε_4_ alleles relative to the risk-neutral ε_3_ allele. In that analysis, pairwise comparisons between the ε_2_ or ε_4_ alleles relative to the ε_3_ allele (e.g. Fig. 3A) were not conducted at each age and sex. Only genes/pathways that were influenced overall by *APOE* genotype, age, sex and interactions between these factors were reported. As our aim is to identify the early changes occurring due to AD-related mutations, we re-analysed only the 3-month brain samples from the Zhao et al. (2020) dataset (i.e. omitting the samples from 12- and 24-month-old mice) to investigate which processes are affected by homozygosity for the ε_2_ or ε_4_ alleles relative to the ε_3_ allele. Hereafter, we refer to these homozygous mice as ‘APOE2’, ‘APOE3’ and ‘APOE4’.

After pre-processing of the APOE-TR RNA-seq data (Figs S15, S16), we performed PCA to visualise the overall similarity between APOE-TR brain transcriptomes. The plot of PC1 against PC2 revealed that samples separated into two distinct clusters of sex across PC2 (Fig. 3B). This suggests that the effect of sex on the murine brain transcriptome is substantial and cannot be ignored in the differential gene expression analysis. Among the male samples, APOE4 samples formed a cluster distinct from the APOE2 and APOE3 samples, suggesting that the APOE4 genotype has a distinct effect on the transcriptome compared to APOE2 relative to APOE3 in males. This was not observed to the same extent in the female samples. However, the male APOE4 and APOE3 samples appeared to have been taken from distinct litters, as implied from the date of birth of each sample (Fig. 3B). This confounds the effect of genotype and complicates interpretation of whether any effects observed in a pairwise comparison between male APOE3 and APOE4 mice are due to *APOE* genotype or litter-of-origin (or, most likely, both). Indeed, χ^2^ tests for independence revealed that there is a highly significant dependence of *APOE* genotype and litter across the entire 3-month-old dataset (χ^2^=82.7, d.f.=20, *P*=1.4×10^−9^), as well as within only male samples (χ^2^=43.1, d.f.=14, *P*=8.2×10^−5^) and within only female samples (χ^2^=39.3, d.f.=14, *P*=3.0×10^−4^). Some litters did not contain sufficient mice to remove the effect (i.e. some coefficients could not be estimated during the generalised linear model fitting procedure due to the design matrix not having full rank). Therefore, we continued the analysis under the assumption of a negligible effect of litter.

To determine which genes were dysregulated in APOE4 mice and APOE2 mice relative to APOE3 mice, we performed a differential gene expression analysis using edgeR (McCarthy et al., 2012; Robinson et al., 2009). Many genes were found to be differentially expressed in each comparison, particularly in male APOE4 mice. Additionally, the biases noted by Zhao et al. (2020) in the original analysis for increased GC content and longer transcript length among differentially expressed genes was also apparent in our analysis (Fig. S17). Therefore, we corrected for these observed biases using conditional quantile normalisation (CQN) (Hansen et al., 2012). After CQN, many genes were identified as differentially expressed in each comparison, and the percentage of GC and gene length biases were decreased (Fig. S18).

We next performed enrichment analysis of the KEGG (Kanehisa and Goto, 2000) and IRE (Hin et al., 2021) gene sets to determine whether changes are observed in APOE4 mice similar to those in our EOFAD-like model zebrafish (Figs S19-S23 and Table S1). We found statistical evidence for significant changes in the expression of oxidative phosphorylation (Fig. 3C) and ribosome (Fig. 3D) gene sets in mice homozygous for the humanised ε_4_ *APOE* allele, consistent with our zebrafish models of EOFAD (although different genes appear to drive the statistical enrichment of the *KEGG_OXIDATIVE_PHOSPHORYLATION* gene set in the two organisms, Fig. S25). Interestingly, we observed highly statistically significant changes in the gene set *ire3_all* only in APOE4 male mice, reminiscent of similar signals in some of the young adult EOFAD-related zebrafish (Barthelson et al., 2020b; Hin et al., 2021; Fig. 1B; Fig. S22C) and supporting the existence of iron dyshomeostasis. These effects were not observed for the AD-protective ε_2_ allele (Fig. 3; Tables S2, S3). However, the effects of *APOE* genotype were highly dependent on the litter-of-origin of the samples, and changes to cell-type proportions were observed in the male APOE4 mice (Fig. S24). Therefore, future replication of this analysis with better-controlled transcriptome data is desirable to confirm that the effects observed are due to the *APOE* genotype.

## DISCUSSION

### Altered gene expression in the oxidative phosphorylation pathway is a transcriptomic signature of genetic variation driving Alzheimer's disease in young adults

Energy production is the most fundamental of cellular activities. Life cannot be sustained without energy, and all other cellular activities depend upon it. The human brain, in particular, has very high energy demands and consumes the majority of the glucose of the body when at rest (reviewed by Zierler, 1999). Within the brain, the majority of energy use is to maintain the Na^+^-K^+^ membrane potential of neurons (Attwell and Laughlin, 2001), and neurons are assisted in meeting these energy demands by support primarily from astrocytes [e.g. via the astrocyte-neuron lactate shuttle (Pellerin and Magistretti, 1994)]. All cells allocate considerable portions of their energy budgets to protein synthesis to maintain their structure and activity (Buttgereit and Brand, 1995). Energy is also required to maintain the low pH and high Ca^2+^ concentration of the lysosome (Christensen et al., 2002), the organelle which mediates the uptake and recycling (autophagy) of cellular structural constituents (e.g. the amino acids for protein synthesis) (reviewed by Yim and Mizushima, 2020). Lysosomes are important for the uptake and recycling of ferrous iron (Yambire et al., 2019), which is essential for oxidative phosphorylation by mitochondria (Oexle et al., 1999). On the lysosomal membrane, mammalian target of rapamycin kinase (mTOR) complexes sense nutrient and energy status to regulate protein synthesis, autophagy and mitochondrial activity (reviewed by Lim and Zoncu, 2016).

The EOFAD genes *PSEN1*, *PSEN2*, *APP* and *SORL1* all encode proteins expressed within the endolysosomal pathway of cells (Andersen et al., 2005; Kawai et al., 1992; Pasternak et al., 2003; Sannerud et al., 2016) and within the mitochondrial associated membranes (MAMs) of the endoplasmic reticulum (Area-Gomez et al., 2009; Lim, 2015). MAMs are responsible for the regulation of ATP production [through Ca^2+^ signalling (Duchen, 1992)], oxidative protein folding (reviewed by Simmen et al., 2010) and the initiation of autophagy (Hamasaki et al., 2013). Interestingly, like EOFAD mutant forms of PSEN1 (Lee et al., 2010) and the C99 fragment of APP (Jiang et al., 2019), the ε4 allele of *APOE* has been shown to affect both lysosomal pH (Prasad and Rao, 2018) and the MAM (Tambini et al., 2016). Our analyses of young adult brain transcriptomes in zebrafish have found that five EOFAD-like mutations in a total of three EOFAD gene orthologues (*psen1*, *psen2* and *sorl1*) all cause statistically significant effects on the expression of genes involved in oxidative phosphorylation, whereas non AD-related mutations in *psen1* and *psen2* do not. Therefore, effects on oxidative phosphorylation are a common, early ‘signature’ of EOFAD. Intriguingly, we previously observed downregulation of the oxidative phosphorylation genes due to heterozygosity for the *psen1*^Q96_K97del^ mutation in whole-zebrafish larvae at 7 days post fertilisation (dpf) (Dong et al., 2021), suggesting changes to mitochondrial function are a very early cellular stress in EOFAD pathogenesis. Additionally, we observed that the ‘semi-dominant’ ε4 LOAD risk allele (Genin et al., 2011), like EOFAD mutations, also affects the expression of genes involved in oxidative phosphorylation and ribosome function in young adult brains. Thus, changes in oxidative phosphorylation (and so energy production) appear to be a common early disturbance associated with both early- and late-onset forms of AD.

The majority of the heterozygous EOFAD-like mutations we have studied in zebrafish cause an overall downregulation of the oxidative phosphorylation gene set in young adult brains relative to wild-type brains (Fig. 1C). Only heterozygosity for the T141_L142delinsMISLISV (reading frame-preserving) mutation of *psen2* has been seen to give overall upregulation of these genes. Another complex yet probable EOFAD-like mutation in *psen2* we have studied in zebrafish, *psen2^S4ter^* (which likely produces Psen2 proteins lacking N-terminal sequences), also showed strong overall upregulation of the oxidative phosphorylation gene set (Jiang et al., 2020); however, that dataset contains technical artefacts that complicate interpretation, so it was not included in the current analysis. Transheterozygosity for mutations in *sorl1* also results in overall upregulation of oxidative phosphorylation genes. We are uncertain as to why this variability in effects on the oxidative phosphorylation gene set occurs. However, for the Presenilins, the single most consistent characteristic of the hundreds of known EOFAD mutations is that they maintain the ability of the genes to produce at least one transcript isoform with the original reading frame [the ‘reading frame preservation rule’ (Jayne et al., 2016)]. This strongly supports that all these mutations act via a dominant gain-of-function molecular mechanism (to interfere with a normal cellular function). Alternatively, it may be that the disruption of this gene set that is consistently observed is a product of both genotype and environmental factors, i.e. the mutant fish may be more or less responsive to environmental variation, such as changes in water quality, microbiome, handling, etc. Additionally, we note that it can be misleading to infer the direction of change in a particular cell activity, such as oxidative phosphorylation, based on the majority behaviour of a (somewhat arbitrarily) defined set of genes. Obviously, actual measurement of, for example, respiratory rates in the zebrafish mutant brains would be needed to establish, with certainty, how the mutations are affecting oxidative phosphorylation. Note, however, that the subtlety of the gene regulatory effects we have observed in the fish models means that discernment of physiological oxygen consumption differences between mutant fish and their wild-type siblings may be challenging. (Simultaneous measurement of differences in the expression levels of the ∼100 genes in the oxidative phosphorylation gene set gives great statistical sensitivity for detection of subtle differences.)

Importantly, male mice homozygous for LOAD risk allele *APOE* ε_4_ showed altered expression of the oxidative phosphorylation gene set, and female APOE4 mice showed a similar trend that did not reach the threshold for statistical significance. In fact, male APOE4 mice appeared to have more disrupted brain transcriptomes than female APOE4 mice (Fig. 3B), including alteration in the abundance of transcripts with IREs in their 3′ UTRs, supporting the possibility of early brain iron dyshomeostasis (as was previously observed in *psen*^*Q96_K97del*/+^ fish and those lacking wild-type *sorl1* function). This was unexpected, as human females are more susceptible to the effects of *APOE* ε_4_ than males (Farrer et al., 1997; Wang et al., 2020). However, other sources of variation (i.e. litter-of-origin, changes to cell-type proportions and possibly transcript length) may be masking the true effects of *APOE* genotype in these mice. Reassuringly, a recent single-cell RNA-seq analysis (in which discrepancies due to differences in cell-type proportions are overcome) of female APOE4 mice showed that expression of oxidative phosphorylation genes is decreased at 12 months of age, particularly in astrocytes. Further dissection of the metabolic phenotype of female APOE4 mice revealed a shift away from oxidative phosphorylation and towards glycolysis for ATP production (Farmer et al., 2021). Remarkably, this result was consistent with observations in human ε_4_-carrying females, who show lower energy expenditure, decreased oxygen consumption and alterations to their plasma metabolomes indicative of increased glycolysis (Farmer et al., 2021).

The changes to gene expression in the oxidative phosphorylation pathway in both EOFAD-like zebrafish and APOE4 mice demonstrates the similarity, at the molecular level, between the cellular effects of genetic variants causing EOFAD and the most significant variant promoting LOAD. The differences in disease onset age between EOFAD and LOAD may be due to the severity of the effects on energy metabolism of the different genetic variants that promote each disease in concert with environmental variables. This is consistent with the fact that classification of AD into these two subtypes appears arbitrary as there is no discernible early age-dependent peak in the population prevalence of dementia (of which AD contributes the majority of cases). The observed molecular pathway similarity between knock-in models of EOFAD and LOAD genetic variation supports the validity and utility of analysing early molecular events in AD pathogenesis using knock-in models in both zebrafish and mice, and that analysis of endogenous EOFAD mutations can contribute information that is valuable for understanding LOAD pathogenesis. The mystery of why GWAS has failed to detect variation in *PSEN1*, *PSEN2* or *APP* in LOAD remains, although some mutations in *PSEN2* and *APP* genes do cause later-onset familial forms of the disease (Cruchaga et al., 2012) and/or show incomplete penetrance (Finckh et al., 2000; Rossor et al., 1996; Sherrington et al., 1996; Thordardottir et al., 2018) or recessive inheritance (Di Fede et al., 2009; Tomiyama et al., 2008).

Brain hypometabolism is a diagnostic criterion for AD and can be visualised by 2-deoxy-2-(^18^F)fluoro-D-glucose positron emission tomography (^18^FDG-PET) (reviewed by Marcus et al., 2014). This technique has been used previously to investigate adults at risk of developing AD (e.g. Ou et al., 2019), whereas blood oxygenation level-dependent (BOLD) functional magnetic resonance imaging (MRI) has identified brain regional changes in activity (and hence energy consumption) in child carriers of the *PSEN1* E280A (‘Paisa’) EOFAD mutation (Quiroz et al., 2015). This suggests that ^18^FDG-PET, or techniques such as dynamic glucose-enhanced MRI (Tolomeo et al., 2018), might enable the screening of individuals to determine the risk of later development of AD and be useful tools for investigating early energy changes in animal models of AD. Indeed, such techniques have been exploited to monitor energy consumption in the brains of transgenic mouse models (Huang et al., 2020; Luo et al., 2012; Poisnel et al., 2012; Tolomeo et al., 2018). However, they have not yet been applied to mice with knock-in EOFAD mutations. The observations of change in *in vivo* brain energy metabolism discussed above are consistent with the observed changes in the expression of genes of the oxidative phosphorylation pathway in the postmortem brains of early and late AD subjects relative to age-matched controls (Manczak et al., 2004). Neuronal cells derived from human induced pluripotent stem cells of LOAD patients also show increased expression of oxidative phosphorylation proteins and oxidative stress (Birnbaum et al., 2018), and neurons derived from a patient carrying the *PSEN1^S170F^* EOFAD mutation showed mitochondrial abnormalities (Li et al., 2020).

Our findings regarding EOFAD mutations in zebrafish were not consistent with findings from our analysis of transcriptome data from homozygous *App^NL-G-F^* mice. However, these transcriptome data were generated from ‘middle-aged’ (12 months old) mice rather than young adults, and the endogenous *App* gene of the mouse was altered with a total of six mutations (three that humanise the sequence of the Aβ region and three EOFAD mutations), motivated by the idea that the more aggregation-prone human Aβ sequence plays a critical role in the pathogenic mechanism of AD. Therefore, it is not directly comparable with our zebrafish EOFAD models, which contain single EOFAD-like mutations within single alleles of endogenous genes. To our knowledge, a transcriptome analysis has not been performed with *App^NL-G-F^* mice at a younger age. However, the expression of genes involved in lysosomal function (*KEGG_LYSOSOME*) was observed to be highly significantly upregulated in homozygous *App^NL-G-F^* brains. This is not unexpected, as acidification of the endolysosomal system is impaired by increased levels of the β-CTF fragment of APP [also known as C99 and generated by β-secretase cleavage of APP (Jiang et al., 2019)]. Increased β-CTF has been observed in the brains of *App^NL-G-F^* mice (Saito et al., 2014). In a mouse model of a lysosomal storage disorder [glycogen storage disease type 2, which most seriously affects muscle (Yambire et al., 2019)], lysosomes failed to become sufficiently acidic, and this resulted in an intracellular ferrous iron deficiency and a pseudohypoxic response, mitochondrial dysfunction and inflammation (Yambire et al., 2019). [Degradation of HIF1-α, the master transcriptional regulator of the cellular response to hypoxia, is dependent on both oxygen and ferrous iron (Ivan et al., 2001).] Additionally, the *App^NL-G-F^* mouse model shows increased levels of Aβ from a young age (Saito et al., 2014), and the deposition of Aβ into plaques was shown to be associated with the increased expression of genes in the complement system in a comprehensive spatial transcriptomics study of aging in the *App^NL-G-F^* mouse model (Chen et al., 2020), providing another avenue for these mutations to trigger inflammation. Mitochondrial dysfunction has not been observed directly in *App^NL-G-F^* mice. However, increased levels of oxidative stress have been observed at 12 months of age (Izumi et al., 2020), suggestive of increased reactive oxygen species that can be generated by dysfunction of mitochondrial respiration. Therefore, we suspect that processes similar to those in our zebrafish models and the APOE4 knock-in mice are being affected in *App^NL-G-F^* mice. However, subtle signs of mitochondrial dysfunction in the transcriptome may be obscured by noise from the strong inflammatory signals in the bulk brain transcriptomic data (as well as confounding influences on the transcriptome analysis, such as litter-of-origin effects).

### mTOR signalling can regulate ribosomal gene set expression

All of the mutations studied have also resulted in changes in the levels of transcripts required for ribosome formation. Protein translation is one of the most energy-costly processes within a cell (Buttgereit and Brand, 1995), and so expression of ribosomal proteins is modulated by the mTOR system, which surveys cellular nutrient status to adjust cellular metabolism (reviewed by Mayer and Grummt, 2006; Zhou et al., 2015). mTOR signalling, which appears to be increased in late-stage AD brains compared to controls (Griffin et al., 2005; Li et al., 2005; Sun et al., 2014), is regulated by growth factors, nutrients, energy levels and stress. In addition to ribosome biogenesis, mTOR signalling plays a role in various other cellular processes also implicated in AD pathogenesis, such as autophagy and lipid metabolism (reviewed by Saxton and Sabatini, 2017).

The mTOR proteins are localised at lysosomes within the mTORC1 and mTORC2 protein complexes (Sancak et al., 2010). Intriguingly, the v-ATPase complex that acidifies the endolysosomal pathway is required for mTORC1 activation (Zoncu et al., 2011). Proper assembly of the v-ATPase at the lysosome requires the PSEN1 protein (and this process is impaired in EOFAD patient fibroblasts) (Lee et al., 2010). Stimulation of mTOR signalling has been observed in response to accumulation of Aβ (Caccamo et al., 2010), and hyperactivation of mTOR is observed in Down's syndrome (in which the dosage of the *APP* gene is increased because it resides on chromosome 21 and early-onset AD is common) (Bordi et al., 2019; Iyer et al., 2014). Intriguingly, Bordi et al. observed that inhibition of mTOR signalling (specifically mTORC1) rescues autophagy and mitophagy defects in the fibroblasts of Down's syndrome individuals (Bordi et al., 2019). Among our transcriptome analyses of AD models, we only observed statistically significant changes to the expression of genes in the KEGG gene set for mTOR signalling in transheterozygous *sorl1* mutants, in *psen1*^Q96_K97del/+^ mutant zebrafish after acute hypoxia exposure, and in both male and female APOE4 mice. However, the majority of regulation of mTOR signalling occurs at the protein level, so that it is perhaps unsurprising that, in the EOFAD mutants, we could only detect significant changes in the transcriptional response to altered mTOR signalling rather than in the mTOR gene set itself.

### Advantages and disadvantages of zebrafish for analysis of genetic variants driving Alzheimer's disease

In a highly sensitive analysis method such as RNA-seq, minimising external sources of variation increases resolving power. Our analysis has revealed that zebrafish can be highly advantageous for transcriptome profiling in the context of RNA-seq, as large numbers of progeny can be produced from a single pair mating, and these can subsequently be raised together in a single aquarium system, thus reducing genetic and environmental variation. This has allowed us to observe subtle effects due to the EOFAD-like mutations we have analysed. In contrast, a female mouse can only birth relatively small litters of 5-10 pups, making it particularly difficult to obtain sufficient numbers of synchronous sibling samples (particularly when genotypes of interest are produced by crossing of heterozygotes). Our re-analysis of the APOE-TR mouse brain transcriptomes was unable to distinguish with great certainty whether the effects we observed were due to *Apoe* genotype or litter-of-origin. Also, information on whether *App^NL-G-F^* mice were littermates was not available, and this required us to assume that the effects of litter were negligible in order to perform the analysis. Another contrast between brain transcriptome analysis in zebrafish compared to mice is the influence of sex. Mouse brain transcriptomes show very significant differences due to sex, whereas sex has a negligible effect on bulk brain transcriptomes from zebrafish (Barthelson et al., 2020b, 2021a,b,c; Drew et al., 2012), and can generally be ignored in a differential expression analysis. We also found evidence for changes to cell-type proportions in both APOE-TR and *App^NL-G-F^* mice, a phenomenon that can create the artefactual appearance of gene expression change. We have not observed cell-type proportion differences in 6-month-old or 24-month-old zebrafish (Barthelson et al., 2020b, 2021a,b,c; Hin et al., 2020, 2021; Fig. S14), possibly because of the resistance to damage of the highly regenerative zebrafish brain (Kroehne et al., 2011). Although this regenerative ability may hinder the use of zebrafish for studying overt neurodegeneration, it can facilitate analysis of young bulk brain transcriptomes before overt pathological processes would be expected.

The advantages of zebrafish for analysing the early effects of EOFAD mutations are countered, occasionally, by disadvantages. The teleost lineage in which zebrafish arose underwent an early whole-genome duplication event (reviewed by Meyer and Van de Peer, 2005), such that many human genes are represented by duplicate ‘co-orthologues’ in zebrafish (e.g. the co-orthologues of *APP* and *APOE* in zebrafish are *appa/appb* and *apoea/apoeb*, respectively). This complicates interpretation of the effects of mutations in these genes. Additionally, zebrafish have never been shown, definitively, to be capable of producing Aβ, a pathological hallmark of AD. The β-secretase (BACE) site of human APP does not appear to be conserved in zebrafish Appa and Appb (Moore et al., 2014). Whether Aβ accumulation is a cause or consequence of AD pathological processes continues as a matter of debate within the AD research community (reviewed by Morris et al., 2018). If zebrafish cannot produce Aβ, then the changes we have observed in the brains of our zebrafish models may illuminate Aβ-independent effects of EOFAD mutations.

### Knock-in models of Alzheimer's disease mutations may model more accurately early AD-associated pathological changes

Knock-in mouse models of single EOFAD mutations were generated 15-20 years ago (Guo et al., 1999; Kawasumi et al., 2004) but their brain transcriptomes have never been analysed in detail. This is probably because these mice showed only very mild cognitive phenotypes and lacked the AD histopathology currently used to define the disease [Aβ deposition and neurofibrillary tangles of tau protein (Jack et al., 2018)]. By expressing multiple mutant forms of EOFAD genes in transgenic mice, Aβ plaques can be detected and cognitive changes observed (reviewed by Esquerda-Canals et al., 2017; Myers and McGonigle, 2019). However, experience of using of many such ‘mouse models of AD’ has shown a lack of correlation of cognitive changes with Aβ levels (Foley et al., 2015) [Aβ levels do not closely correlate with cognitive changes in humans either (Giannakopoulos et al., 2003)], and transcriptome analysis of their brains has shown little to no concordance with transcriptomes from postmortem AD brains, or between the models themselves (Hargis and Blalock, 2017). In two papers, Saito et al. (2014, 2016) described phenotypic disparities between transgenic and *APP* EOFAD mutation knock-in mouse models. In the 2016 paper, they went so far as to declare that, “We recently estimated using single App knock-in mice that accumulate amyloid β peptide without transgene overexpression that 60% of the phenotypes observed in Alzheimer's model mice overexpressing mutant amyloid precursor protein (APP) or APP and presenilin are artifacts (Saito et al., 2014). The current study further supports this estimate by invalidating key results from papers that were published in Cell. These findings suggest that more than 3000 publications based on APP and APP/PS overexpression must be reevaluated.”

Nevertheless, since 2016, thousands more papers have been published using transgenic mouse models of AD. In this light, we were surprised to find that *App^NL-G-F^* homozygous mice display a young adult brain transcriptome that is more severely disturbed than in the triple transgenic 3xTg-AD model – although that apparent disturbance is likely somewhat artefactual and due to changes in the relative proportions of different cell types in the model. As mentioned above, changes to cell-type proportions are not observed in our zebrafish models (Barthelson et al., 2020b, 2021b,c; Fig. S14).

Frustration with the difficulties of exploiting both transgenic and knock-in models of EOFAD mutations in mice has contributed to the drive for examining knock-in mouse models of LOAD risk variants, such as now conducted by the Model Organism Development and Evaluation for Late-Onset Alzheimer's Disease Consortium (Oblak et al., 2020). The brain transcriptome similarities seen between our single mutation heterozygous EOFAD mutation-like knock-in zebrafish models and the knock-in *APOE* ε4 mice strongly support the informative value of these models, and imply that heterozygous EOFAD mutation knock-in mouse models offer a path forward, particularly in understanding the earliest molecular events that lead to AD.

## MATERIALS AND METHODS

### Analysis of knock-in zebrafish models of EOFAD

For analysis of 6-month-old and 24-month-old genome-edited Tübingen zebrafish (sample sizes and sexes are indicated in Table 1 and Figs S1-S6), we obtained the differential gene expression analysis outputs and harmonic mean *P*-values (statistical significance of gene sets) from each individual analysis (see Barthelson et al., 2020b, 2021a,b,c). For these analyses, differential gene expression analysis was performed using edgeR (Robinson et al., 2009), and enrichment analysis was performed by calculation of the harmonic mean *P*-value (Wilson, 2019) of the raw *P*-values of three methods of ranked-list-based enrichment analyses: fry (Wu et al., 2010), camera (Wu and Smyth, 2012) and GSEA (Subramanian et al., 2005) [as implemented in the fgsea R package (Sergushichev, 2016 preprint)]. We used the harmonic mean *P*-value to determine the overall significance of changes to gene expression within gene sets because this method does not assume that component *P*-values are independent (Wilson, 2019). We have previously validated the use of the harmonic mean *P*-value on simulated RNA-seq datasets (Barthelson et al., 2020b). We considered a gene set to be significantly altered if the false discovery rate (FDR)-adjusted harmonic mean *P*-value remained below 0.05. The gene sets used for enrichment analysis were the KEGG (Kanehisa and Goto, 2000) gene sets, so that any changes to gene expression in any of 186 biological pathways/processes could be determined. Additionally, we used our recently defined IRE gene sets (Hin et al., 2021) to test for evidence of iron dyshomeostasis. For the K97fs and Q96_K97del analyses, enrichment analysis was not performed on the KEGG gene sets in the original analyses. Therefore, we performed the enrichment analysis as described above for these datasets. For the K97fs analysis, we obtained the gene-level counts and the results of the differential gene expression analysis described by Hin et al. (2020a) from www.github.com/UofABioinformaticsHub/k97fsZebrafishAnalysis. For the Q96_K97del analysis, we obtained the gene-level counts and the results of the differential gene expression analysis described by Hin et al. (2020b) from the first author of the cited paper. Note that, for each zebrafish analysis, the sample size was usually *n*=6 zebrafish per genotype, based on our previous calculation that this sample size should give ∼70% power to detect the majority of expressed transcripts in a zebrafish brain transcriptome at a fold change of >2 and at an FDR of 0.05 (Barthelson et al., 2020b).

### APP^NL-G-F^ microarray re-analysis

The raw .CEL files were obtained from GEO and analysed with R (https://www.r-project.org/). Pre-processing was performed using the RMA (Irizarry et al., 2003) method as implemented in the oligo package (Carvalho and Irizarry, 2010). We omitted any probesets that contained a median log2 intensity value of <3.5 (lowly expressed) and also any probesets assigned to multiple genes. Differential gene expression analysis was performed using limma (Ritchie et al., 2015), specifying pairwise contrasts between the *App^NL-G-F^* homozygous mice or the 3xTg homozygous mice with their respective controls by using a contrasts matrix. We considered a probeset to be differentially expressed in each contrast if the FDR-adjusted *P*-value was <0.05. For over-representation of the KEGG and IRE gene sets within the differentially expressed genes, we used kegga (Young et al., 2010). We also performed ranked-list-based enrichment analysis using the harmonic mean *P*-value as described for the zebrafish analyses.

### APOE-TR RNA-seq re-analysis

We obtained the raw fastq files for the entire APOE-TR RNA-seq experiment from the AD Knowledge Portal (accession number syn20808171, https://adknowledgeportal.synapse.org/). The raw reads were first processed using AdapterRemoval (version 2.2.1) (Schubert et al., 2016), with following options selected: --trimns, --trimqualities, --minquality 30 and --minlength 35*.* Then, the trimmed reads were aligned to the *Mus musculus* genome (Ensembl GRCm38, release 98) using STAR (version 2.7.0) (Dobin et al., 2013), using default parameters to generate .bam files. These bam files were then sorted and indexed using SAMtools (version 1.10) (Li et al., 2009). The gene expression counts matrix was generated from the bam files using featureCounts (version 1.5.2) (Liao et al., 2014). We only counted the number of reads that uniquely aligned to, strictly, exons with a mapping quality of at least ten to predict expression levels of genes in each sample.

We then imported the output from featureCounts (Liao et al., 2014) for analysis with R. We first omitted genes that were lowly expressed (and are uninformative for differential expression analysis). We considered a gene to be lowly expressed if it contained, at most, 2 counts per million in 8 or more of the 24 samples we analysed. We also assessed whether the sex of each sample was correctly classified by examining the expression of genes that are located on the Y chromosome. Three samples appeared to be classified incorrectly and were subsequently corrected (Fig. S16).

To determine which genes were dysregulated in APOE4 and APOE2 mice relative to APOE3, we performed a differential gene expression analysis using a generalised linear model and likelihood ratio tests using edgeR (McCarthy et al., 2012; Robinson et al., 2009). We chose a design matrix that specified the *APOE* genotype and sex of each sample. The contrasts matrix was specified to compare the effect of APOE2 or APOE4 relative to APOE3 in males and in females. In this analysis, we considered a gene to be differentially expressed if the FDR-adjusted *P*-value was <0.05. A bias for longer transcript length and higher GC content percentage was observed in this dataset. Therefore, we corrected for this bias using CQN (Hansen et al., 2012). We calculated the average transcript length per gene, and a weighted (by transcript length) average GC content percentage per gene, as input to CQN to produce the offset to correct for the bias. This offset was then included in an additional generalised linear model and likelihood ratio tests in edgeR with the same design and contrast matrices. For over-representation of the KEGG and IRE gene sets within the differentially expressed genes, we used goseq (Young et al., 2010), specifying average transcript length to generate the probability weighting function, which corrects for the probability that a gene is classified as differentially expressed based on its transcript length (average transcript length per gene) alone. We also performed ranked-list-based enrichment analysis as described for the zebrafish analysis.

Visualisation of gene expression data throughout this analysis was performed using ggplot2 (Wickham, 2016), pheatmap (available at https://CRAN.R-project.org/package=pheatmap) and pathview (Luo et al., 2017). The code used to perform the analysis in this study can be found at www.github.com/karissa-b/AD-signature.

## Supplementary Material

Supplementary information

## References

[DMM049187C1] Alzheimer, A. (1906). Über einen eigenartigen schweren erkrankungsprozeβ der hirnrincle. *Neurol Cent.* 25, 1134.

[DMM049187C2] Andersen, O. M., Reiche, J., Schmidt, V., Gotthardt, M., Spoelgen, R., Behlke, J., von Arnim, C. A., Breiderhoff, T., Jansen, P., Wu, X. et al. (2005). Neuronal sorting protein-related receptor sorLA/LR11 regulates processing of the amyloid precursor protein. *Proc. Natl. Acad. Sci. U.S.A.* 102, 13461-13466. 10.1073/pnas.0503689102 16174740 PMC1224625

[DMM049187C3] Area-Gomez, E., de Groof, A. J., Boldogh, I., Bird, T. D., Gibson, G. E., Koehler, C. M., Yu, W. H., Duff, K. E., Yaffe, M. P., Pon, L. A. et al. (2009). Presenilins are enriched in endoplasmic reticulum membranes associated with mitochondria. *Am. J. Pathol.* 175, 1810-1816. 10.2353/ajpath.2009.090219 19834068 PMC2774047

[DMM049187C4] Attwell, D. and Laughlin, S. B. (2001). An energy budget for signaling in the grey matter of the brain. *J. Cereb. Blood Flow Metab.* 21, 1133-1145. 10.1097/00004647-200110000-00001 11598490

[DMM049187C129] Ayodele, T., Rogaeva, E., Kurup, J. T., Beecham, G. and Reitz, C. (2021). Early-onset Alzheimer's disease: What Is missing in research? *Curr. Neurol. Neurosci. Rep.* 21, 4. 10.1007/s11910-020-01090-y 33464407 PMC7815616

[DMM049187C5] Barthelson, K., Newman, M. and Lardelli, M. (2020a). Sorting out the role of the *Sortilin-related receptor 1* in Alzheimer's disease. *J. Alzheimers Dis. Rep.* 4, 123-140. 10.3233/ADR-200177 32587946 PMC7306921

[DMM049187C6] Barthelson, K., Pederson, S. M., Newman, M. and Lardelli, M. (2020b). Brain transcriptome analysis reveals subtle effects on mitochondrial function and iron homeostasis of mutations in the *SORL1* gene implicated in early onset familial Alzheimer's disease. *Mol. Brain* 13, 142. 10.1186/s13041-020-00681-7 33076949 PMC7570131

[DMM049187C7] Barthelson, K., Dong, Y., Newman, M. and Lardelli, M. (2021a). PRESENILIN 1 mutations causing early-onset familial Alzheimer's disease or familial acne inversa differ in their effects on genes facilitating energy metabolism and signal transduction. *J. Alzheimers Dis.* 82, 327-347. 10.3233/JAD-210128 34024832

[DMM049187C8] Barthelson, K., Pederson, S. M., Newman, M., Jiang, H. and Lardelli, M. (2021b). In-frame and frameshift mutations in zebrafish *Presenilin 2* affect different cellular functions in young adult brains. *J. Alzheimers Dis. Rep.* 5, 395-404. 10.3233/ADR-200279 34189411 PMC8203281

[DMM049187C9] Barthelson, K., Pederson, S. M., Newman, M. and Lardelli, M. (2021c). Brain transcriptome analysis of a protein-truncating mutation in *Sortilin-related receptor 1* associated with early-onset familial Alzheimer's disease indicates early effects on mitochondrial and ribosome function. *J. Alzheimers Dis.* 79, 1105-1119. 10.3233/JAD-201383 33386808

[DMM049187C10] Bertram, L. and Tanzi, R. E. (2012). The genetics of Alzheimer's disease. *Prog. Mol. Biol. Transl. Sci.* 107, 79. 10.1016/B978-0-12-385883-2.00008-4 22482448

[DMM049187C11] Birnbaum, J. H., Wanner, D., Gietl, A. F., Saake, A., Kündig, T. M., Hock, C., Nitsch, R. M. and Tackenberg, C. (2018). Oxidative stress and altered mitochondrial protein expression in the absence of amyloid-β and tau pathology in iPSC-derived neurons from sporadic Alzheimer's disease patients. *Stem Cell Res.* 27, 121-130. 10.1016/j.scr.2018.01.019 29414602

[DMM049187C12] Blennow, K., de Leon, M. J. and Zetterberg, H. (2006). Alzheimer's disease. *The Lancet* 368, 387-403. 10.1016/S0140-6736(06)69113-7 16876668

[DMM049187C13] Bordi, M., Darji, S., Sato, Y., Mellén, M., Berg, M. J., Kumar, A., Jiang, Y. and Nixon, R. A. (2019). mTOR hyperactivation in Down Syndrome underlies deficits in autophagy induction, autophagosome formation, and mitophagy. *Cell Death & Disease* 10, 563. 10.1038/s41419-019-1752-5 31332166 PMC6646359

[DMM049187C14] Buttgereit, F. and Brand, M. D. (1995). A hierarchy of ATP-consuming processes in mammalian cells. *Biochem. J.* 312, 163-167. 10.1042/bj3120163 7492307 PMC1136240

[DMM049187C15] Caccamo, A., Majumder, S., Richardson, A., Strong, R. and Oddo, S. (2010). Molecular interplay between mammalian target of rapamycin (mTOR), amyloid-beta, and Tau: effects on cognitive impairments. *J. Biol. Chem.* 285, 13107-13120. 10.1074/jbc.M110.100420 20178983 PMC2857107

[DMM049187C16] Carvalho, B. S. and Irizarry, R. A. (2010). A framework for oligonucleotide microarray preprocessing. *Bioinformatics* 26, 2363-2367. 10.1093/bioinformatics/btq431 20688976 PMC2944196

[DMM049187C17] Castillo, E., Leon, J., Mazzei, G., Abolhassani, N., Haruyama, N., Saito, T., Saido, T., Hokama, M., Iwaki, T., Ohara, T. et al. (2017). Comparative profiling of cortical gene expression in Alzheimer's disease patients and mouse models demonstrates a link between amyloidosis and neuroinflammation. *Sci. Rep.* 7, 17762. 10.1038/s41598-017-17999-3 29259249 PMC5736730

[DMM049187C18] Chen, W.-T., Lu, A., Craessaerts, K., Pavie, B., Sala Frigerio, C., Corthout, N., Qian, X., Laláková, J., Kühnemund, M., Voytyuk, I. et al. (2020). Spatial transcriptomics and *in situ* sequencing to study Alzheimer's disease. *Cell* 182, 976-991.e19. 10.1016/j.cell.2020.06.038 32702314

[DMM049187C19] Christensen, K. A., Myers, J. T. and Swanson, J. A. (2002). pH-dependent regulation of lysosomal calcium in macrophages. *J. Cell Sci.* 115, 599. 10.1242/jcs.115.3.599 11861766

[DMM049187C20] Corder, E. H., Saunders, A. M., Strittmatter, W. J., Schmechel, D. E., Gaskell, P. C., Small, G. W., Roses, A. D., Haines, J. L. and Pericak-Vance, M. A. (1993). Gene dose of apolipoprotein E type 4 allele and the risk of Alzheimer's disease in late onset families. *Science* 261, 921-923. 10.1126/science.8346443 8346443

[DMM049187C21] Cruchaga, C., Haller, G., Chakraverty, S., Mayo, K., Vallania, F. L. M., Mitra, R. D., Faber, K., Williamson, J., Bird, T., Diaz-Arrastia, R. et al. (2012). Rare variants in APP, PSEN1 and PSEN2 increase risk for AD in late-onset Alzheimer's disease families. *PLoS ONE* 7, e31039. 10.1371/journal.pone.0031039 22312439 PMC3270040

[DMM049187C22] DeTure, M. A. and Dickson, D. W. (2019). The neuropathological diagnosis of Alzheimer's disease. *Mol. Neurodegener.* 14, 32. 10.1186/s13024-019-0333-5 31375134 PMC6679484

[DMM049187C23] Di Fede, G., Catania, M., Morbin, M., Rossi, G., Suardi, S., Mazzoleni, G., Merlin, M., Giovagnoli, A. R., Prioni, S. and Erbetta, A. (2009). A recessive mutation in the APP gene with dominant-negative effect on amyloidogenesis. *Science* 323, 1473-1477. 10.1126/science.1168979 19286555 PMC2728497

[DMM049187C24] Dobin, A., Davis, C. A., Schlesinger, F., Drenkow, J., Zaleski, C., Jha, S., Batut, P., Chaisson, M. and Gingeras, T. R. (2013). STAR: ultrafast universal RNA-seq aligner. *Bioinformatics* 29, 15-21. 10.1093/bioinformatics/bts635 23104886 PMC3530905

[DMM049187C25] Dong, Y., Newman, M., Pederson, S. M., Barthelson, K., Hin, N. and Lardelli, M. (2021). Transcriptome analyses of 7-day-old zebrafish larvae possessing a familial Alzheimer's disease-like mutation in *psen1* indicate effects on oxidative phosphorylation, ECM and MCM functions, and iron homeostasis. *BMC Genomics* 22, 211. 10.1186/s12864-021-07509-1 33761877 PMC7992352

[DMM049187C26] Drew, R. E., Settles, M. L., Churchill, E. J., Williams, S. M., Balli, S. and Robison, B. D. (2012). Brain transcriptome variation among behaviorally distinct strains of zebrafish (*Danio rerio*). *BMC Genomics* 13, 323. 10.1186/1471-2164-13-323 22817472 PMC3434030

[DMM049187C27] Duchen, M. R. (1992). Ca(^2+^)-dependent changes in the mitochondrial energetics in single dissociated mouse sensory neurons. *Biochem. J.* 283, 41-50. 10.1042/bj2830041 1373604 PMC1130990

[DMM049187C28] Esquerda-Canals, G., Montoliu-Gaya, L., Güell-Bosch, J. and Villegas, S. (2017). Mouse models of Alzheimer's disease. *J. Alzheimers Dis.* 57, 1171-1183. 10.3233/JAD-170045 28304309

[DMM049187C29] Farmer, B. C., Williams, H. C., Devanney, N. A., Piron, M. A., Nation, G. K., Carter, D. J., Walsh, A. E., Khanal, R., Young, L. E. A., Kluemper, J. C. et al. (2021). APOΕ_4_ lowers energy expenditure in females and impairs glucose oxidation by increasing flux through aerobic glycolysis. *Molecular Neurodegeneration* 16, 62. 10.1186/s13024-021-00483-y 34488832 PMC8420022

[DMM049187C30] Farrer, L. A., Cupples, L. A., Haines, J. L., Hyman, B., Kukull, W. A., Mayeux, R., Myers, R. H., Pericak-Vance, M. A., Risch, N. and van Duijn, C. M. (1997). Effects of age, sex, and ethnicity on the association between apolipoprotein E genotype and Alzheimer disease. A meta-analysis. APOE and Alzheimer Disease Meta Analysis Consortium. *JAMA* 278, 1349-1356. 9343467

[DMM049187C31] Finckh, U., Alberici, A., Antoniazzi, M., Benussi, L., Fedi, V., Giannini, C., Gal, A., Nitsch, R. M. and Binetti, G. (2000). Variable expression of familial Alzheimer disease associated with presenilin 2 mutation M239I. *Neurology* 54, 2006-2008. 10.1212/WNL.54.10.2006 10822446

[DMM049187C32] Foley, A. M., Ammar, Z. M., Lee, R. H. and Mitchell, C. S. (2015). Systematic review of the relationship between amyloid-β levels and measures of transgenic mouse cognitive deficit in Alzheimer's disease. *J. Alzheimers Dis.* 44, 787-795. 10.3233/JAD-142208 25362040 PMC4346318

[DMM049187C33] Genin, E., Hannequin, D., Wallon, D., Sleegers, K., Hiltunen, M., Combarros, O., Bullido, M. J., Engelborghs, S., De Deyn, P., Berr, C. et al. (2011). APOE and Alzheimer disease: a major gene with semi-dominant inheritance. *Mol. Psychiatry* 16, 903-907. 10.1038/mp.2011.52 21556001 PMC3162068

[DMM049187C34] Giannakopoulos, P., Herrmann, F. R., Bussière, T., Bouras, C., Kövari, E., Perl, D. P., Morrison, J. H., Gold, G. and Hof, P. R. (2003). Tangle and neuron numbers, but not amyloid load, predict cognitive status in Alzheimer's disease. *Neurology* 60, 1495-1500. 10.1212/01.WNL.0000063311.58879.01 12743238

[DMM049187C35] Griffin, R. J., Moloney, A., Kelliher, M., Johnston, J. A., Ravid, R., Dockery, P., O'Connor, R. and O'Neill, C. (2005). Activation of Akt/PKB, increased phosphorylation of Akt substrates and loss and altered distribution of Akt and PTEN are features of Alzheimer's disease pathology. *J. Neurochem.* 93, 105-117. 10.1111/j.1471-4159.2004.02949.x 15773910

[DMM049187C36] Guo, Q., Fu, W., Sopher, B. L., Miller, M. W., Ware, C. B., Martin, G. M. and Mattson, M. P. (1999). Increased vulnerability of hippocampal neurons to excitotoxic necrosis in presenilin-1 mutant knock-in mice. *Nat. Med.* 5, 101-106. 10.1038/4789 9883847

[DMM049187C37] Hamasaki, M., Furuta, N., Matsuda, A., Nezu, A., Yamamoto, A., Fujita, N., Oomori, H., Noda, T., Haraguchi, T., Hiraoka, Y. et al. (2013). Autophagosomes form at ER-mitochondria contact sites. *Nature* 495, 389-393. 10.1038/nature11910 23455425

[DMM049187C38] Hansen, K. D., Irizarry, R. A. and Wu, Z. (2012). Removing technical variability in RNA-seq data using conditional quantile normalization. *Biostatistics* 13, 204-216. 10.1093/biostatistics/kxr054 22285995 PMC3297825

[DMM049187C39] Hargis, K. E. and Blalock, E. M. (2017). Transcriptional signatures of brain aging and Alzheimer's disease: What are our rodent models telling us? *Behav. Brain Res.* 322, 311-328. 10.1016/j.bbr.2016.05.007 27155503 PMC5533609

[DMM049187C40] Hin, N., Newman, M., Kaslin, J., Douek, A. M., Lumsden, A., Nik, S. H. M., Dong, Y., Zhou, X.-F., Mañucat-Tan, N. B., Ludington, A. et al. (2020). Accelerated brain aging towards transcriptional inversion in a zebrafish model of the K115fs mutation of human PSEN2. *PLoS One* 15, e0227258. 31978074 10.1371/journal.pone.0227258 PMC6980398

[DMM049187C41] Hin, N., Newman, M., Pederson, S. and Lardelli, M. (2021). Iron responsive element-mediated responses to iron dyshomeostasis in Alzheimer's disease. *J. Alzheimers Dis.* 84, 1597-1630. 10.3233/JAD-210200 34719489

[DMM049187C42] Huang, J., van Zijl, P. C. M., Han, X., Dong, C. M., Cheng, G. W. Y., Tse, K.-H., Knutsson, L., Chen, L., Lai, J. H. C., Wu, E. X. et al. (2020). Altered d-glucose in brain parenchyma and cerebrospinal fluid of early Alzheimer's disease detected by dynamic glucose-enhanced MRI. *Sci. Adv.* 6, eaba3884. 10.1126/sciadv.aba3884 32426510 PMC7220384

[DMM049187C43] Irizarry, R. A., Bolstad, B. M., Collin, F., Cope, L. M., Hobbs, B. and Speed, T. P. (2003). Summaries of Affymetrix GeneChip probe level data. *Nucleic Acids Res.* 31, e15. 10.1093/nar/gng015 12582260 PMC150247

[DMM049187C44] Iturria-Medina, Y., Sotero, R., Toussaint, P., Mateos-Pérez, J., Evans, A. and Initiative, A. S. D. N. (2016). Early role of vascular dysregulation on late-onset Alzheimer's disease based on multifactorial data-driven analysis. *Nat. Commun.* 7, 11934. doi:10.1038/ncomms11934 27327500 10.1038/ncomms11934 PMC4919512

[DMM049187C45] Ivan, M., Kondo, K., Yang, H., Kim, W., Valiando, J., Ohh, M., Salic, A., Asara, J. M., Lane, W. S. and Kaelin , Jr, W. G. (2001). HIFα Targeted for VHL-Mediated Destruction by Proline Hydroxylation: Implications for O_2_ Sensing. *Science* 292, 464-468. 10.1126/science.1059817 11292862

[DMM049187C46] Iyer, A. M., van Scheppingen, J., Milenkovic, I., Anink, J. J., Adle-Biassette, H., Kovacs, G. G. and Aronica, E. (2014). mTOR hyperactivation in Down syndrome hippocampus appears early during development. *J. Neuropathol. Exp. Neurol.* 73, 671-683. 10.1097/NEN.0000000000000083 24918639

[DMM049187C47] Izumi, H., Sato, K., Kojima, K., Saito, T., Saido, T. C. and Fukunaga, K. (2020). Oral glutathione administration inhibits the oxidative stress and the inflammatory responses in AppNL–G-F/NL–G-F knock-in mice. *Neuropharmacology* 168, 108026. 10.1016/j.neuropharm.2020.108026 32130977

[DMM049187C48] Jack, C. R., Jr., Bennett, D. A., Blennow, K., Carrillo, M. C., Dunn, B., Haeberlein, S. B., Holtzman, D. M., Jagust, W., Jessen, F., Karlawish, J. et al. (2018). NIA-AA Research Framework: Toward a biological definition of Alzheimer's disease. *Alzheimers Dement.* 14, 535-562. 10.1016/j.jalz.2018.02.018 29653606 PMC5958625

[DMM049187C49] Jansen, I. E., Savage, J. E., Watanabe, K., Bryois, J., Williams, D. M., Steinberg, S., Sealock, J., Karlsson, I. K., Hägg, S., Athanasiu, L. et al. (2019). Genome-wide meta-analysis identifies new loci and functional pathways influencing Alzheimer's disease risk. *Nat. Genet.* 51, 404-413. 10.1038/s41588-018-0311-9 30617256 PMC6836675

[DMM049187C50] Jayne, T., Newman, M., Verdile, G., Sutherland, G., Munch, G., Musgrave, I., Moussavi Nik, S. H. and Lardelli, M. (2016). Evidence for and against a pathogenic role of reduced γ-secretase activity in familial Alzheimer's disease. *J. Alzheimers Dis.* 52, 781-799. 10.3233/JAD-151186 27060961

[DMM049187C51] Jiang, Y., Sato, Y., Im, E., Berg, M., Bordi, M., Darji, S., Kumar, A., Mohan, P. S., Bandyopadhyay, U., Diaz, A. et al. (2019). Lysosomal dysfunction in Down syndrome Is APP-dependent and mediated by APP-βCTF (C99). *J. Neurosci.* 39, 5255. 10.1523/JNEUROSCI.0578-19.2019 31043483 PMC6607756

[DMM049187C52] Jiang, H., Pederson, S. M., Newman, M., Dong, Y., Barthelson, K. and Lardelli, M. (2020). Transcriptome analysis indicates dominant effects on ribosome and mitochondrial function of a premature termination codon mutation in the zebrafish gene *psen2*. *PLoS ONE* 15, e0232559. 10.1371/journal.pone.0232559 32658922 PMC7357760

[DMM049187C53] Kanehisa, M. and Goto, S. (2000). KEGG: Kyoto Encyclopedia of Genes and Genomes. *Nucleic Acids Res.* 28, 27-30. 10.1093/nar/28.1.27 10592173 PMC102409

[DMM049187C54] Kawai, M., Cras, P., Richey, P., Tabaton, M., Lowery, D. E., Gonzalez-DeWhitt, P. A., Greenberg, B. D., Gambetti, P. and Perry, G. (1992). Subcellular localization of amyloid precursor protein in senile plaques of Alzheimer's disease. *Am. J. Pathol.* 140, 947-958. 1562053 PMC1886361

[DMM049187C55] Kawasumi, M., Chiba, T., Yamada, M., Miyamae-Kaneko, M., Matsuoka, M., Nakahara, J., Tomita, T., Iwatsubo, T., Kato, S., Aiso, S. et al. (2004). Targeted introduction of V642I mutation in amyloid precursor protein gene causes functional abnormality resembling early stage of Alzheimer's disease in aged mice. *Eur. J. Neurosci.* 19, 2826-2838. 10.1111/j.0953-816X.2004.03397.x 15147316

[DMM049187C57] Kroehne, V., Freudenreich, D., Hans, S., Kaslin, J. and Brand, M. (2011). Regeneration of the adult zebrafish brain from neurogenic radial glia-type progenitors. *Development* 138, 4831. 10.1242/dev.072587 22007133

[DMM049187C58] Kunkle, B. W., Grenier-Boley, B., Sims, R., Bis, J. C., Damotte, V., Naj, A. C., Boland, A., Vronskaya, M., Van Der Lee, S. J., Amlie-Wolf, A. et al. (2019). Genetic meta-analysis of diagnosed Alzheimer's disease identifies new risk loci and implicates Aβ, tau, immunity and lipid processing. *Nat. Genet.* 51, 414-430. 10.1038/s41588-019-0358-2 30820047 PMC6463297

[DMM049187C59] Lambert, J.-C., Ibrahim-Verbaas, C. A., Harold, D., Naj, A. C., Sims, R., Bellenguez, C., Jun, G., DeStefano, A. L., Bis, J. C., Beecham, G. W. et al. (2013). Meta-analysis of 74,046 individuals identifies 11 new susceptibility loci for Alzheimer's disease. *Nat. Genet.* 45, 1452. 10.1038/ng.2802 24162737 PMC3896259

[DMM049187C60] Lee, J.-H., Yu, W. H., Kumar, A., Lee, S., Mohan, P. S., Peterhoff, C. M., Wolfe, D. M., Martinez-Vicente, M., Massey, A. C., Sovak, G. et al. (2010). Lysosomal proteolysis and autophagy require presenilin 1 and are disrupted by Alzheimer-related PS1 mutations. *Cell* 141, 1146-1158. 10.1016/j.cell.2010.05.008 20541250 PMC3647462

[DMM049187C61] Li, X., Alafuzoff, I., Soininen, H., Winblad, B. and Pei, J.-J. (2005). Levels of mTOR and its downstream targets 4E-BP1, eEF2, and eEF2 kinase in relationships with tau in Alzheimer's disease brain. *FEBS J.* 272, 4211-4220. 10.1111/j.1742-4658.2005.04833.x 16098202

[DMM049187C62] Li, H., Handsaker, B., Wysoker, A., Fennell, T., Ruan, J., Homer, N., Marth, G., Abecasis, G. and Durbin, R. (2009). The sequence alignment/map format and SAMtools. *Bioinformatics* 25, 2078-2079. 10.1093/bioinformatics/btp352 19505943 PMC2723002

[DMM049187C63] Li, L., Kim, H. J., Roh, J. H., Kim, M., Koh, W., Kim, Y., Heo, H., Chung, J., Nakanishi, M., Yoon, T. et al. (2020). Pathological manifestation of the induced pluripotent stem cell-derived cortical neurons from an early-onset Alzheimer's disease patient carrying a presenilin-1 mutation (S170F). *Cell Prolif.* 53, e12798. 32216003 10.1111/cpr.12798 PMC7162796

[DMM049187C64] Liao, Y., Smyth, G. K. and Shi, W. (2014). featureCounts: an efficient general purpose program for assigning sequence reads to genomic features. *Bioinformatics* 30, 923-930. 10.1093/bioinformatics/btt656 24227677

[DMM049187C65] Lim, A. H. L. (2015). Analysis of the subcellular localization of proteins implicated in Alzheimer's disease. Genetics and evolution. *PhD thesis*, pp. 235, University of Adelaide, Adelaide, Australia.

[DMM049187C66] Lim, C.-Y. and Zoncu, R. (2016). The lysosome as a command-and-control center for cellular metabolism. *J. Cell Biol.* 214, 653-664. 10.1083/jcb.201607005 27621362 PMC5021098

[DMM049187C67] Lumsden, A. L., Rogers, J. T., Majd, S., Newman, M., Sutherland, G. T., Verdile, G. and Lardelli, M. (2018). Dysregulation of neuronal iron homeostasis as an alternative unifying effect of mutations causing familial Alzheimer's disease. *Front. Neurosci.* 12, 533. 10.3389/fnins.2018.00533 30150923 PMC6099262

[DMM049187C68] Luo, F., Rustay, N. R., Ebert, U., Hradil, V. P., Cole, T. B., Llano, D. A., Mudd, S. R., Zhang, Y., Fox, G. B. and Day, M. (2012). Characterization of 7- and 19-month-old Tg2576 mice using multimodal in vivo imaging: Limitations as a translatable model of Alzheimer's disease. *Neurobiol. Aging* 33, 933-944. 10.1016/j.neurobiolaging.2010.08.005 20961663

[DMM049187C69] Luo, W., Pant, G., Bhavnasi, Y. K., Blanchard, S. G., Jr. and Brouwer, C. (2017). Pathview Web: user friendly pathway visualization and data integration. *Nucleic Acids Res.* 45, W501-W508. 10.1093/nar/gkx372 28482075 PMC5570256

[DMM049187C70] Manczak, M., Park, B. S., Jung, Y. and Reddy, P. H. (2004). Differential expression of oxidative phosphorylation genes in patients with Alzheimer's disease. *Neuromolecular Med.* 5, 147-162. 10.1385/NMM:5:2:147 15075441

[DMM049187C71] Marcus, C., Mena, E. and Subramaniam, R. M. (2014). Brain PET in the diagnosis of Alzheimer's disease. *Clin. Nuc. Med.* 39, e413-422. 10.1097/RLU.0000000000000547 PMC4332800 25199063

[DMM049187C72] Masters, C. L., Bateman, R., Blennow, K., Rowe, C. C., Sperling, R. A. and Cummings, J. L. (2015). Alzheimer's disease. *Nat. Rev. Dis. Primers* 1, 15056. 10.1038/nrdp.2015.56 27188934

[DMM049187C73] Mayer, C. and Grummt, I. (2006). Ribosome biogenesis and cell growth: mTOR coordinates transcription by all three classes of nuclear RNA polymerases. *Oncogene* 25, 6384-6391. 10.1038/sj.onc.1209883 17041624

[DMM049187C74] McCarthy, D. J., Chen, Y. and Smyth, G. K. (2012). Differential expression analysis of multifactor RNA-Seq experiments with respect to biological variation. *Nucleic Acids Res.* 40, 4288-4297. 10.1093/nar/gks042 22287627 PMC3378882

[DMM049187C75] Meyer, A. and Van de Peer, Y. (2005). From 2R to 3R: evidence for a fish-specific genome duplication (FSGD). *BioEssays* 27, 937-945. 10.1002/bies.20293 16108068

[DMM049187C76] Moore, D. B., Gillentine, M. A., Botezatu, N. M., Wilson, K. A., Benson, A. E. and Langeland, J. A. (2014). Asynchronous evolutionary origins of Aβ and BACE1. *Mol. Biol. Evol.* 31, 696-702. 10.1093/molbev/mst262 24361992 PMC3935185

[DMM049187C77] Morris, G. P., Clark, I. A. and Vissel, B. (2018). Questions concerning the role of amyloid-β in the definition, aetiology and diagnosis of Alzheimer's disease. *Acta Neuropathol.* 136, 663-689. 30349969 10.1007/s00401-018-1918-8 PMC6208728

[DMM049187C78] Moussavi Nik, S. H., Newman, M., Wilson, L., Ebrahimie, E., Wells, S., Musgrave, I., Verdile, G., Martins, R. N. and Lardelli, M. (2015). Alzheimer's disease-related peptide PS2V plays ancient, conserved roles in suppression of the unfolded protein response under hypoxia and stimulation of γ-secretase activity. *Hum. Mol. Genet.* 24, 3662-3678. 25814654 10.1093/hmg/ddv110

[DMM049187C79] Myers, A. and McGonigle, P. (2019). Overview of transgenic mouse models for Alzheimer's disease. *Curr Protoc Neurosci* 89, e81. 10.1002/cpns.81 31532917

[DMM049187C80] Newman, M., Hin, N., Pederson, S. and Lardelli, M. (2019). Brain transcriptome analysis of a familial Alzheimer's disease-like mutation in the zebrafish presenilin 1 gene implies effects on energy production. *Mol. Brain* 12, 43. 10.1186/s13041-019-0467-y 31053140 PMC6500017

[DMM049187C81] Oblak, A. L., Forner, S., Territo, P. R., Sasner, M., Carter, G. W., Howell, G. R., Sukoff-Rizzo, S. J., Logsdon, B. A., Mangravite, L. M., Mortazavi, A. et al. (2020). Model organism development and evaluation for late-onset Alzheimer's disease: MODEL-AD. *Alzheimer's & Dementia: Translational Research & Clinical Interventions* 6, e12110. 33283040 10.1002/trc2.12110 PMC7683958

[DMM049187C82] Oddo, S., Caccamo, A., Shepherd, J. D., Murphy, M. P., Golde, T. E., Kayed, R., Metherate, R., Mattson, M. P., Akbari, Y. and LaFerla, F. M. (2003). Triple-transgenic model of Alzheimer's disease with plaques and tangles: intracellular Abeta and synaptic dysfunction. *Neuron* 39, 409-421. 10.1016/S0896-6273(03)00434-3 12895417

[DMM049187C83] Oexle, H., Gnaiger, E. and Weiss, G. (1999). Iron-dependent changes in cellular energy metabolism: influence on citric acid cycle and oxidative phosphorylation. *Biochim. Biophys. Acta* 1413, 99-107. 10556622 10.1016/s0005-2728(99)00088-2

[DMM049187C84] Ou, Y.-N., Xu, W., Li, J.-Q., Guo, Y., Cui, M., Chen, K.-L., Huang, Y.-Y., Dong, Q., Tan, L., Yu, J.-T. et al. (2019). FDG-PET as an independent biomarker for Alzheimer's biological diagnosis: a longitudinal study. *Alzheimer's Research & Therapy* 11, 57. 10.1186/s13195-019-0512-1 PMC6599313 31253185

[DMM049187C85] Pasternak, S. H., Bagshaw, R. D., Guiral, M., Zhang, S., Ackerley, C. A., Pak, B. J., Callahan, J. W. and Mahuran, D. J. (2003). Presenilin-1, nicastrin, amyloid precursor protein, and gamma-secretase activity are co-localized in the lysosomal membrane. *J. Biol. Chem.* 278, 26687-26694. 10.1074/jbc.M304009200 12736250

[DMM049187C86] Pellerin, L. and Magistretti, P. J. (1994). Glutamate uptake into astrocytes stimulates aerobic glycolysis: a mechanism coupling neuronal activity to glucose utilization. *Proc. Natl. Acad. Sci. U.S.A.* 91, 10625-10629. 7938003 10.1073/pnas.91.22.10625 PMC45074

[DMM049187C87] Poisnel, G., Hérard, A.-S., El Tayara, N. E. T., Bourrin, E., Volk, A., Kober, F., Delatour, B., Delzescaux, T., Debeir, T., Rooney, T. et al. (2012). Increased regional cerebral glucose uptake in an APP/PS1 model of Alzheimer's disease. *Neurobiol. Aging* 33, 1995-2005. 10.1016/j.neurobiolaging.2011.09.026 22079157 PMC3666917

[DMM049187C88] Pottier, C., Hannequin, D., Coutant, S., Rovelet-Lecrux, A., Wallon, D., Rousseau, S., Legallic, S., Paquet, C., Bombois, S., Pariente, J. et al. (2012). High frequency of potentially pathogenic SORL1 mutations in autosomal dominant early-onset Alzheimer disease. *Mol. Psychiatry* 17, 875-879. 10.1038/mp.2012.15 22472873

[DMM049187C89] Prasad, H. and Rao, R. (2018). Amyloid clearance defect in ApoE4 astrocytes is reversed by epigenetic correction of endosomal pH. *Proc. Natl Acad. Sci. USA* 115, E6640. 29946028 10.1073/pnas.1801612115 PMC6048470

[DMM049187C90] Quiroz, Y. T., Schultz, A. P., Chen, K., Protas, H. D., Brickhouse, M., Fleisher, A. S., Langbaum, J. B., Thiyyagura, P., Fagan, A. M., Shah, A. R. et al. (2015). Brain Imaging and Blood Biomarker Abnormalities in Children With Autosomal Dominant Alzheimer Disease. 72, 912. 10.1001/jamaneurol.2015.1099 PMC4625544 26121081

[DMM049187C92] Ritchie, M. E., Phipson, B., Wu, D., Hu, Y., Law, C. W., Shi, W. and Smyth, G. K. (2015). limma powers differential expression analyses for RNA-sequencing and microarray studies. *Nucleic Acids Res.* 43, e47. 10.1093/nar/gkv007 25605792 PMC4402510

[DMM049187C93] Robinson, M. D., McCarthy, D. J. and Smyth, G. K. (2009). edgeR: a Bioconductor package for differential expression analysis of digital gene expression data. *Bioinformatics* 26, 139-140. 10.1093/bioinformatics/btp616 19910308 PMC2796818

[DMM049187C94] Rossor, M., Fox, N., Beck, J., Campbell, T. and Collinge, J. (1996). Incomplete penetrance of familial Alzheimer's disease in a pedigree with a novel presenilin-1 gene mutation. *The Lancet* 347, 1560. 10.1016/S0140-6736(96)90715-1 8684135

[DMM049187C95] Saito, T., Matsuba, Y., Mihira, N., Takano, J., Nilsson, P., Itohara, S., Iwata, N. and Saido, T. C. (2014). Single App knock-in mouse models of Alzheimer's disease. *Nat. Neurosci.* 17, 661-663. 10.1038/nn.3697 24728269

[DMM049187C96] Saito, T., Matsuba, Y., Yamazaki, N., Hashimoto, S. and Saido, T. C. (2016). Calpain Activation in Alzheimer's Model Mice Is an Artifact of APP and Presenilin Overexpression. *J. Neurosci.* 36, 9933. 10.1523/JNEUROSCI.1907-16.2016 27656030 PMC5030353

[DMM049187C97] Sancak, Y., Bar-Peled, L., Zoncu, R., Markhard, A. L., Nada, S. and Sabatini, D. M. (2010). Ragulator-Rag Complex Targets mTORC1 to the Lysosomal Surface and Is Necessary for Its Activation by Amino Acids. *Cell* 141, 290-303. 10.1016/j.cell.2010.02.024 20381137 PMC3024592

[DMM049187C98] Sannerud, R., Esselens, C., Ejsmont, P., Mattera, R., Rochin, L., Tharkeshwar, A. K., De Baets, G., De Wever, V., Habets, R., Baert, V. et al. (2016). Restricted location of PSEN2/γ-secretase determines substrate specificity and generates an intracellular Aβ pool. *Cell* 166, 193-208. 10.1016/j.cell.2016.05.020 27293189 PMC7439524

[DMM049187C99] Sato, N., Hori, O., Yamaguchi, A., Lambert, J.-C., Chartier-Harlin, M.-C., Robinson, P. A., Delacourte, A., Schmidt, A. M., Furuyama, T., Imaizumi, K. et al. (1999). A novel presenilin-2 splice variant in human Alzheimer's disease brain tissue. *J. Neurochem.* 72, 2498-2505. 10.1046/j.1471-4159.1999.0722498.x 10349860

[DMM049187C100] Saunders, A. M., Schmader, K., Breitner, J. C., Benson, M. D., Brown, W. T., Goldfarb, L., Goldgaber, D., Manwaring, M. G., Szymanski, M. H., McCown, N. et al. (1993). Apolipoprotein E epsilon 4 allele distributions in late-onset Alzheimer's disease and in other amyloid-forming diseases. *Lancet* 342, 710-711. 8103823 10.1016/0140-6736(93)91709-u

[DMM049187C101] Saxton, R. A. and Sabatini, D. M. (2017). mTOR signaling in growth, metabolism, and disease. *Cell* 168, 960-976. 10.1016/j.cell.2017.02.004 28283069 PMC5394987

[DMM049187C102] Schubert, M., Lindgreen, S. and Orlando, L. (2016). AdapterRemoval v2: rapid adapter trimming, identification, and read merging. *BMC Research Notes* 9, 88. 10.1186/s13104-016-1900-2 26868221 PMC4751634

[DMM049187C103] Sergushichev, A. A. (2016). An algorithm for fast preranked gene set enrichment analysis using cumulative statistic calculation. *bioRxiv* 060012.

[DMM049187C104] Sherrington, R., Froelich, S., Sorbi, S., Campion, D., Chi, H., Rogaeva, E. A., Levesque, G., Rogaev, E. I., Lin, C., Liang, Y. et al. (1996). Alzheimer's disease associated with mutations in presenilin 2 is rare and variably penetrant. *Hum. Mol. Genet.* 5, 985-988. 10.1093/hmg/5.7.985 8817335

[DMM049187C105] Simmen, T., Lynes, E. M., Gesson, K. and Thomas, G. (2010). Oxidative protein folding in the endoplasmic reticulum: Tight links to the mitochondria-associated membrane (MAM). *Biochimica et Biophysica Acta (BBA) - Biomembranes* 1798, 1465-1473. 20430008 10.1016/j.bbamem.2010.04.009 PMC2885528

[DMM049187C106] Sims, R., Hill, M. and Williams, J. (2020). The multiplex model of the genetics of Alzheimer's disease. *Nat. Neurosci.* 23, 311-322. 10.1038/s41593-020-0599-5 32112059

[DMM049187C107] Subramanian, A., Tamayo, P., Mootha, V. K., Mukherjee, S., Ebert, B. L., Gillette, M. A., Paulovich, A., Pomeroy, S. L., Golub, T. R., Lander, E. S. et al. (2005). Gene set enrichment analysis: A knowledge-based approach for interpreting genome-wide expression profiles. *Proc. Natl Acad. Sci. USA* 102, 15545. 16199517 10.1073/pnas.0506580102 PMC1239896

[DMM049187C108] Sullivan, P. M., Mezdour, H., Aratani, Y., Knouff, C., Najib, J., Reddick, R. L., Quarfordt, S. H. and Maeda, N. (1997). Targeted replacement of the mouse apolipoprotein E gene with the common human APOE3 allele enhances diet-induced hypercholesterolemia and atherosclerosis. *J. Biol. Chem.* 272, 17972-17980. 10.1074/jbc.272.29.17972 9218423

[DMM049187C109] Sun, Y.-X., Ji, X., Mao, X., Xie, L., Jia, J., Galvan, V., Greenberg, D. A. and Jin, K. (2014). Differential activation of mTOR complex 1 signaling in human brain with mild to severe Alzheimer's disease. *J, Alzheimers Dis.* 38, 437-444. 10.3233/JAD-131124 23979023

[DMM049187C110] Tambini, M. D., Pera, M., Kanter, E., Yang, H., Guardia-Laguarta, C., Holtzman, D., Sulzer, D., Area-Gomez, E. and Schon, E. A. (2016). ApoE4 upregulates the activity of mitochondria-associated ER membranes. *EMBO Rep.* 17, 27-36. 10.15252/embr.201540614 26564908 PMC4718413

[DMM049187C111] Tellechea, P., Pujol, N., Esteve-Belloch, P., Echeveste, B., García-Eulate, M. R., Arbizu, J. and Riverol, M. (2018). Early- and late-onset Alzheimer disease: Are they the same entity? *Neurologia* 33, 244-253. 10.1016/j.nrl.2015.08.002 26546285

[DMM049187C113] Thonberg, H., Chiang, H.-H., Lilius, L., Forsell, C., Lindström, A.-K., Johansson, C., Björkström, J., Thordardottir, S., Sleegers, K., Van Broeckhoven, C. et al. (2017). Identification and description of three families with familial Alzheimer disease that segregate variants in the SORL1 gene. *Acta Neuropathologica Communications* 5, 43. 28595629 10.1186/s40478-017-0441-9 PMC5465543

[DMM049187C114] Thordardottir, S., Rodriguez-Vieitez, E., Almkvist, O., Ferreira, D., Saint-Aubert, L., Kinhult-Ståhlbom, A., Thonberg, H., Schöll, M., Westman, E., Wall, A. et al. (2018). Reduced penetrance of the PSEN1 H163Y autosomal dominant Alzheimer mutation: a 22-year follow-up study. *Alzheimer's Research & Therapy* 10, 45. 10.1186/s13195-018-0374-y PMC5944151 29747683

[DMM049187C115] Tolomeo, D., Micotti, E., Serra, S. C., Chappell, M., Snellman, A. and Forloni, G. (2018). Chemical exchange saturation transfer MRI shows low cerebral 2-deoxy-D-glucose uptake in a model of Alzheimer's Disease. *Sci. Rep.* 8, 9576. 10.1038/s41598-018-27839-7 29934551 PMC6015016

[DMM049187C116] Tomiyama, T., Nagata, T., Shimada, H., Teraoka, R., Fukushima, A., Kanemitsu, H., Takuma, H., Kuwano, R., Imagawa, M., Ataka, S. et al. (2008). A new amyloid β variant favoring oligomerization in Alzheimer's-type dementia. *Ann. Neurol.* 63, 377-387. 10.1002/ana.21321 18300294

[DMM049187C117] Wang, Y.-T., Kang, M. S., Therriault, J., Pascoal, T. A., Lussier, F. Z., Savard, M., Benedet, A. L., Tissot, C., Arias, J. F., Gauthier, S. et al. (2020). APOE4 packs a punch in women: Sex-specific vulnerability for tau and neuroinflammation. *Alzheimer's & Dementia* 16, e045098.

[DMM049187C118] Wickham, H. (2016). *ggplot2: Elegant Graphics for Data Analysis*. Springer-Verlag, New York.

[DMM049187C119] Wilson, D. J. (2019). The harmonic mean p-value for combining dependent tests. *Proc. Natl Acad. Sci. USA* 116, 1195. 30610179 10.1073/pnas.1814092116 PMC6347718

[DMM049187C120] Wu, D. and Smyth, G. K. (2012). Camera: a competitive gene set test accounting for inter-gene correlation. *Nucleic Acids Res.* 40, e133. 10.1093/nar/gks461 22638577 PMC3458527

[DMM049187C121] Wu, D., Lim, E., Vaillant, F., Asselin-Labat, M.-L., Visvader, J. E. and Smyth, G. K. (2010). ROAST: rotation gene set tests for complex microarray experiments. *Bioinformatics* 26, 2176-2182. 10.1093/bioinformatics/btq401 20610611 PMC2922896

[DMM049187C122] Yambire, K. F., Rostosky, C., Watanabe, T., Pacheu-Grau, D., Torres-Odio, S., Sanchez-Guerrero, A., Senderovich, O., Meyron-Holtz, E. G., Milosevic, I., Frahm, J. et al. (2019). Impaired lysosomal acidification triggers iron deficiency and inflammation in vivo. *Elife* 8, e51031. 31793879 10.7554/eLife.51031 PMC6917501

[DMM049187C123] Yim, W. W.-Y. and Mizushima, N. (2020). Lysosome biology in autophagy. *Cell Discovery* 6, 6. 10.1038/s41421-020-0141-7 32047650 PMC7010707

[DMM049187C124] Young, M. D., Wakefield, M. J., Smyth, G. K. and Oshlack, A. (2010). Gene ontology analysis for RNA-seq: accounting for selection bias. *Genome Biol.* 11, R14. 10.1186/gb-2010-11-2-r14 20132535 PMC2872874

[DMM049187C125] Zhao, N., Ren, Y., Yamazaki, Y., Qiao, W., Li, F., Felton, L. M., Mahmoudiandehkordi, S., Kueider-Paisley, A., Sonoustoun, B., Arnold, M. et al. (2020). Alzheimer's risk factors age, APOE genotype, and sex drive distinct molecular pathways. *Neuron* 106, 727-742.e6. 32199103 10.1016/j.neuron.2020.02.034 PMC7388065

[DMM049187C126] Zhou, X., Liao, W.-J., Liao, J.-M., Liao, P. and Lu, H. (2015). Ribosomal proteins: functions beyond the ribosome. *J. Mol. Cell Biol.* 7, 92-104. 10.1093/jmcb/mjv014 25735597 PMC4481666

[DMM049187C127] Zierler, K. (1999). Whole body glucose metabolism. *American Journal of Physiology-Endocrinology and Metabolism* 276, E409-E426. 10.1152/ajpendo.1999.276.3.E409 10070005

[DMM049187C128] Zoncu, R., Bar-Peled, L., Efeyan, A., Wang, S., Sancak, Y. and Sabatini, D. M. (2011). mTORC1 senses lysosomal amino acids through an inside-out mechanism that requires the vacuolar H(+)-ATPase. *Science* 334, 678. 10.1126/science.1207056 22053050 PMC3211112

