# Supplementary material for: Brain transcriptomes of zebrafish and mouse Alzheimer's disease knock-in models imply early disrupted energy metabolism
Source: Dis Model Mech. 2022 Jan 26;15(1):dmm049187. doi: 10.1242/dmm.049187 (PMC8807579; doi:10.1242/dmm.049187)
Supplement: Supplementary information [file dmm-15-049187-s1.pdf]

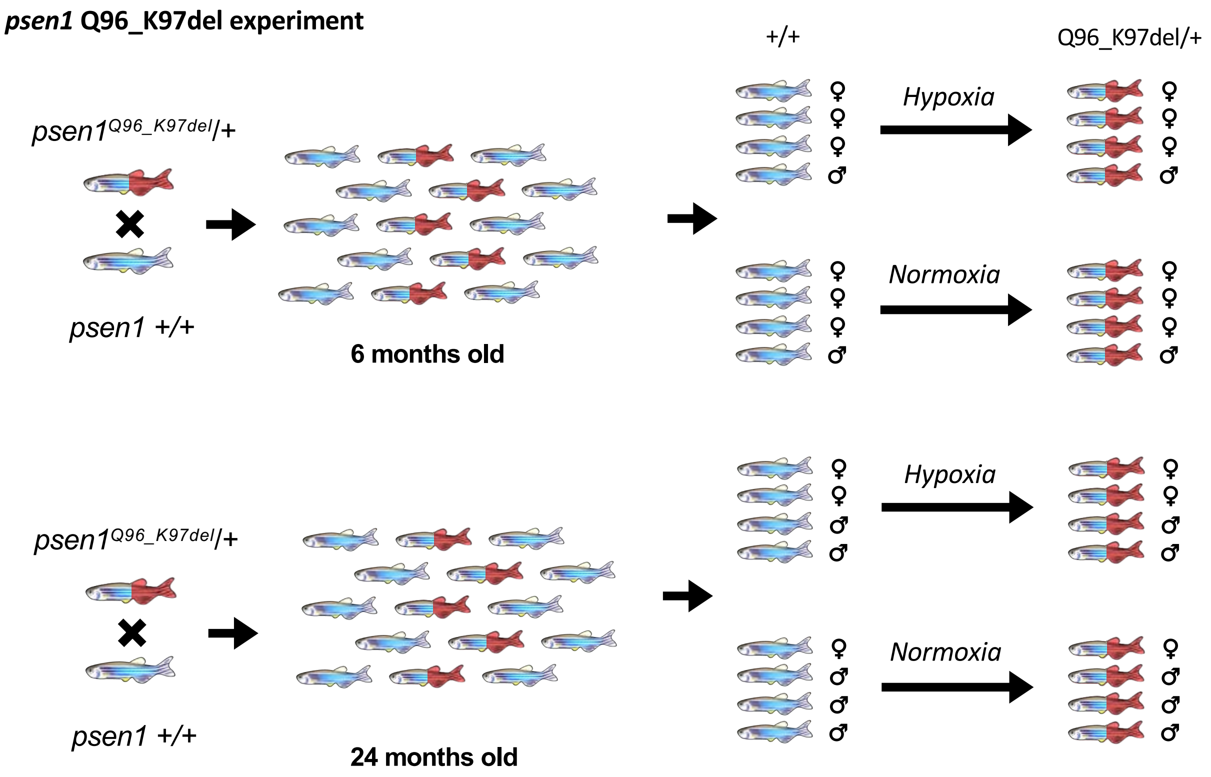

Fig. S1. Two families of zebrafish were generated by mating a wild type fish with a fish heterozygous for the Q96_K97del mutation of *psen1*, resulting in families of fish either heterozygous for the Q96_K97del mutation, or wild type. These families were raised together in single tanks until 6 or 24 months of age. Then, subsets of the families were genotyped using allele-specific polymerase chain reactions (PCRs), followed by hypoxia treatment. Then fish were sacrificed and n = 4 fish per genotype and treatment were subject to RNA-seq analysis.

Disease Models & Mechanisms • Supplementary information

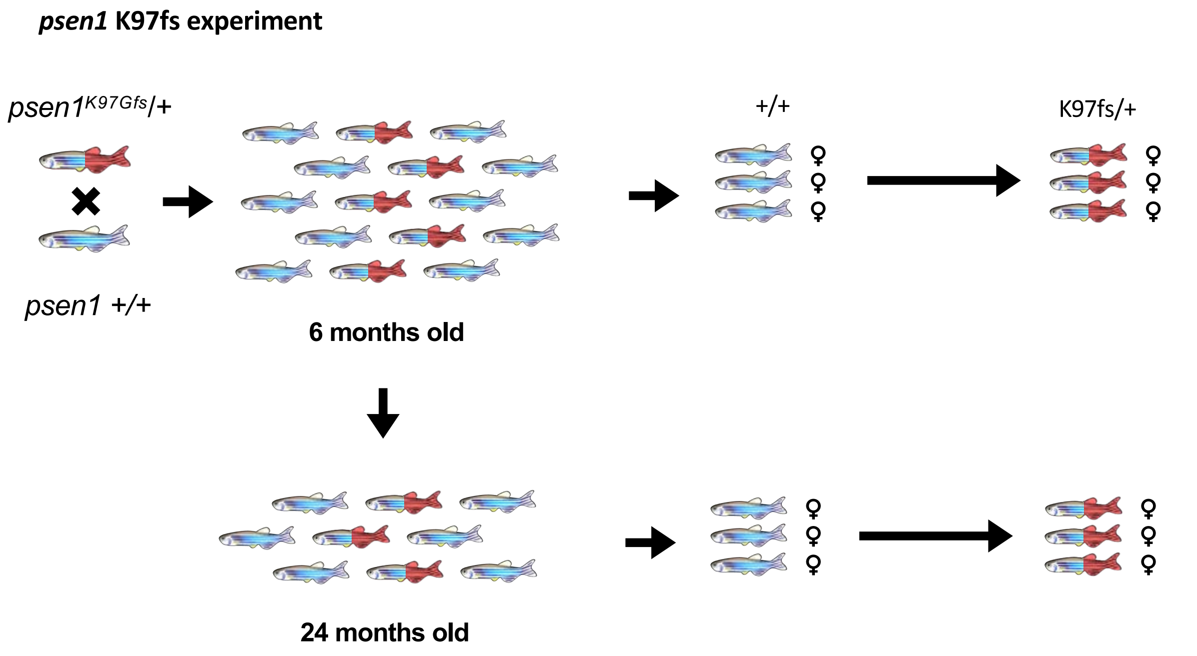

Fig. S2. A wild type fish was mated with a fish heterozygous for the K97Gfs mutation of *psen1*, resulting in a family fish either heterozygous for the K97Gfs mutation, or wild type. This family of fish was raised together in a single tank until 6 months of age. Then, a subset of the family were genotyped using allele-specific polymerase chain reactions (PCRs), then fish were sacrificed and brains were removed for RNA-seq. The remaining fish in the tank were allowed to develop until 24 months of age where this was repeated to generate the aged samples for RNA-seq.

psen1 **T428del vs W233fs experiment**

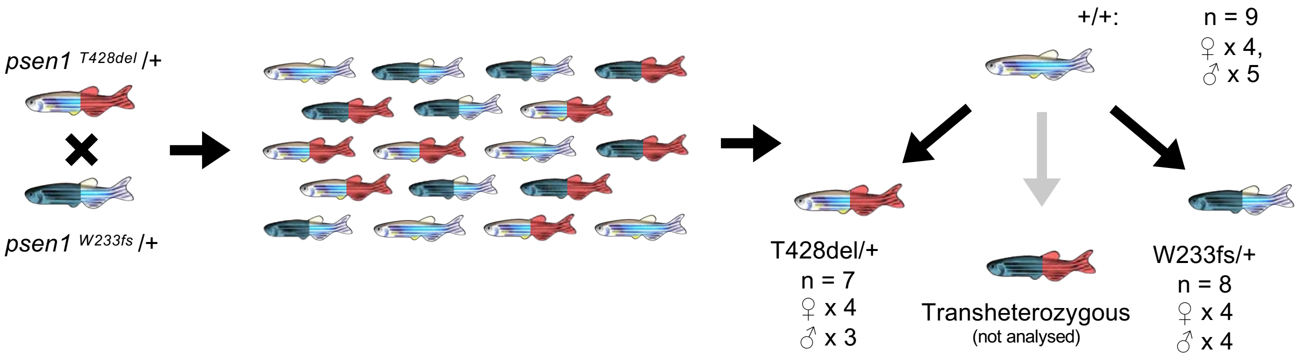

Fig. S3. A fish heterozygous for the T428del (EOfAD-like) mutation of *psen1* was mated with a fish heterozygous for the W233fs mutation (similar to the P242fs mutation of human *PSEN1* causative for familial acne inversa) to generate a family of sibling fish with four possible *psen1* genotypes. This family was raised together in the same tank until 6 months of age, at which time 50 fish were randomly selected and sacrificed in a loose ice slurry. Fish were genotyped after sacrifice by allele specific PCRs. Then n = 8 fish per genotype (4 females and 4 males) were subjected to RNA-seq analysis. During the RNA-seq analysis, one T428del/+ fish was identified to be incorrectly genotyped and was re-classified as wild type.

Disease Models & Mechanisms • Supplementary information

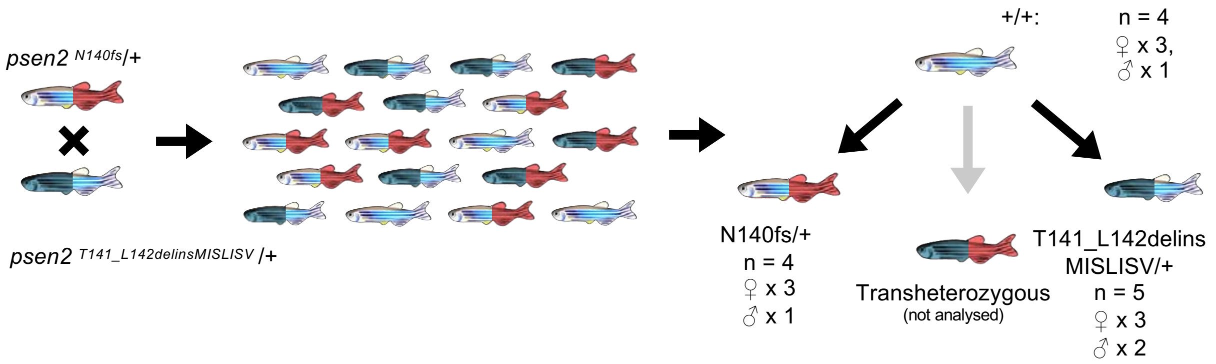

Fig. S4. A fish heterozygous for the N140fs (not EOfAD-like) mutation of *psen2* was mated with a fish heterozygous for the T141_L142delinsMISLISV (EOfAD-like) mutation of *psen2* to generate a family of sibling fish with four possible *psen2* genotypes. This family was raised together in the same tank until 6 months of age, at which time 24 fish were randomly selected and sacrificed in a loose ice slurry (to allow for n = 5 of each genotype in the RNA-seq analysis). Fish were genotyped after sacrifice by allele specific PCRs. Then n = 5 fish per genotype (3 females and 2 males) were subjected to RNA-seq analysis. During the RNA-seq analysis, one wild type fish was an obvious outlier and was omitted from the rest of the analysis, and one N140s/+ sample has been incorrectly genotyped and was also omitted.

Disease Models & Mechanisms • Supplementary information

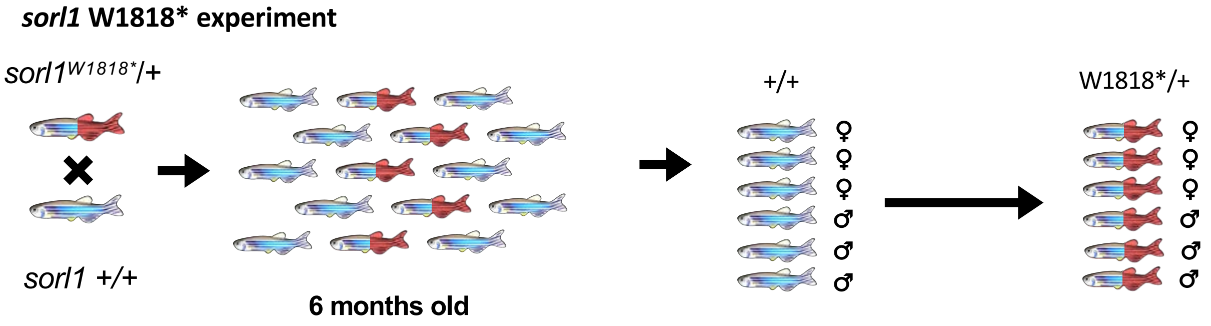

Fig. S5. A fish heterozygous for the W1818* (EOfAD-like) mutation of *sorl1* was mated with a wild type fish to generate a family of sibling fish with two possible *sorl1* genotypes. This family was raised together in the same tank until 6 months of age, at which time 20 fish were randomly selected and sacrificed in a loose ice slurry (to allow for n = 6 of each genotype in the RNA-seq analysis). Fish were genotyped after sacrifice by allele specific PCRs. Then n = 3 fish per genotype and sex were subjected to RNA-seq analysis.

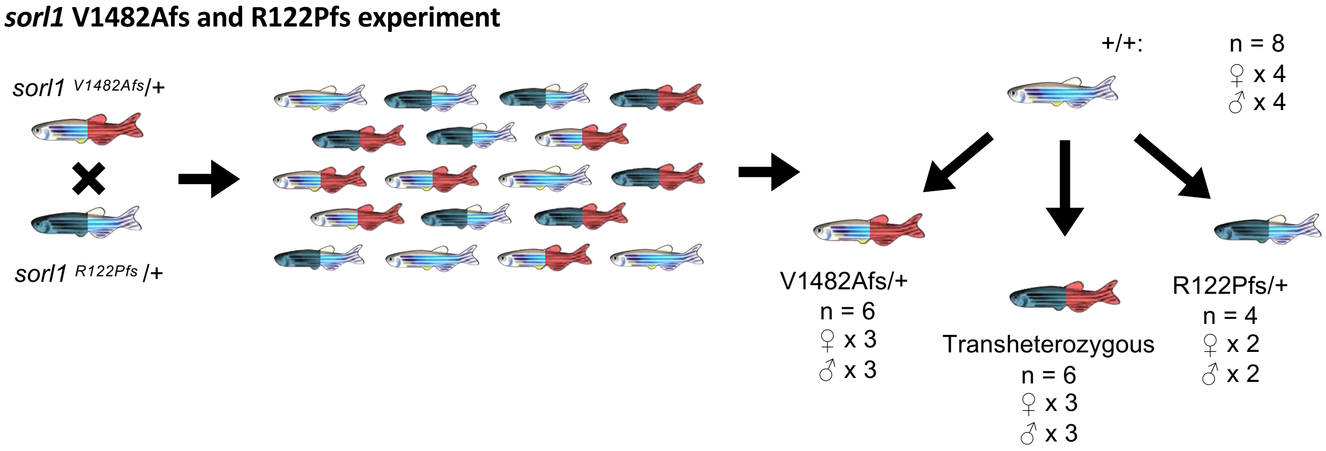

Fig. S6. A fish heterozygous for the V1482Afs (EOfAD-like) mutation of *sorl1* was mated with a fish heterozygous for the R122Pfs mutation to generate a family of sibling fish with four possible *sorl1* genotypes. This family was raised together in the same tank until 6 months of age, at which time 50 fish were randomly selected and sacrificed in a loose ice slurry (to allow for n = 6 of each genotype in the RNA-seq analysis). Fish were genotyped after sacrifice by allele specific PCRs. During the RNA-seq analysis, two R122Pfs/+ fish were identified to be incorrectly genotyped and were re-classified as wild type.

Disease Models & Mechanisms • Supplementary information

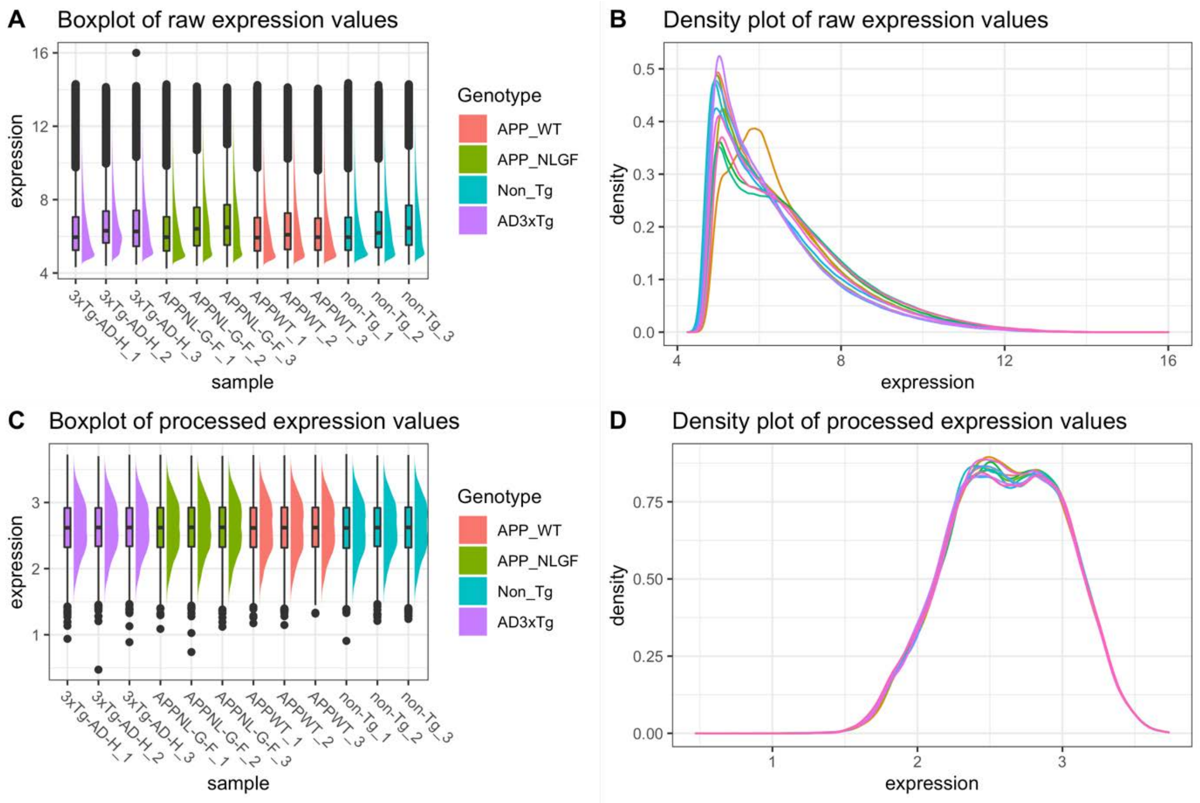

Fig. S7. A) Boxplot and **B)** density plot of the raw intensity data of GSE92926. **C)** Boxplot and **D)** density plot of the intensity data after rma normalisation and filtering for lowly expressed and multi-mapping probes.

Disease Models & Mechanisms • Supplementary information

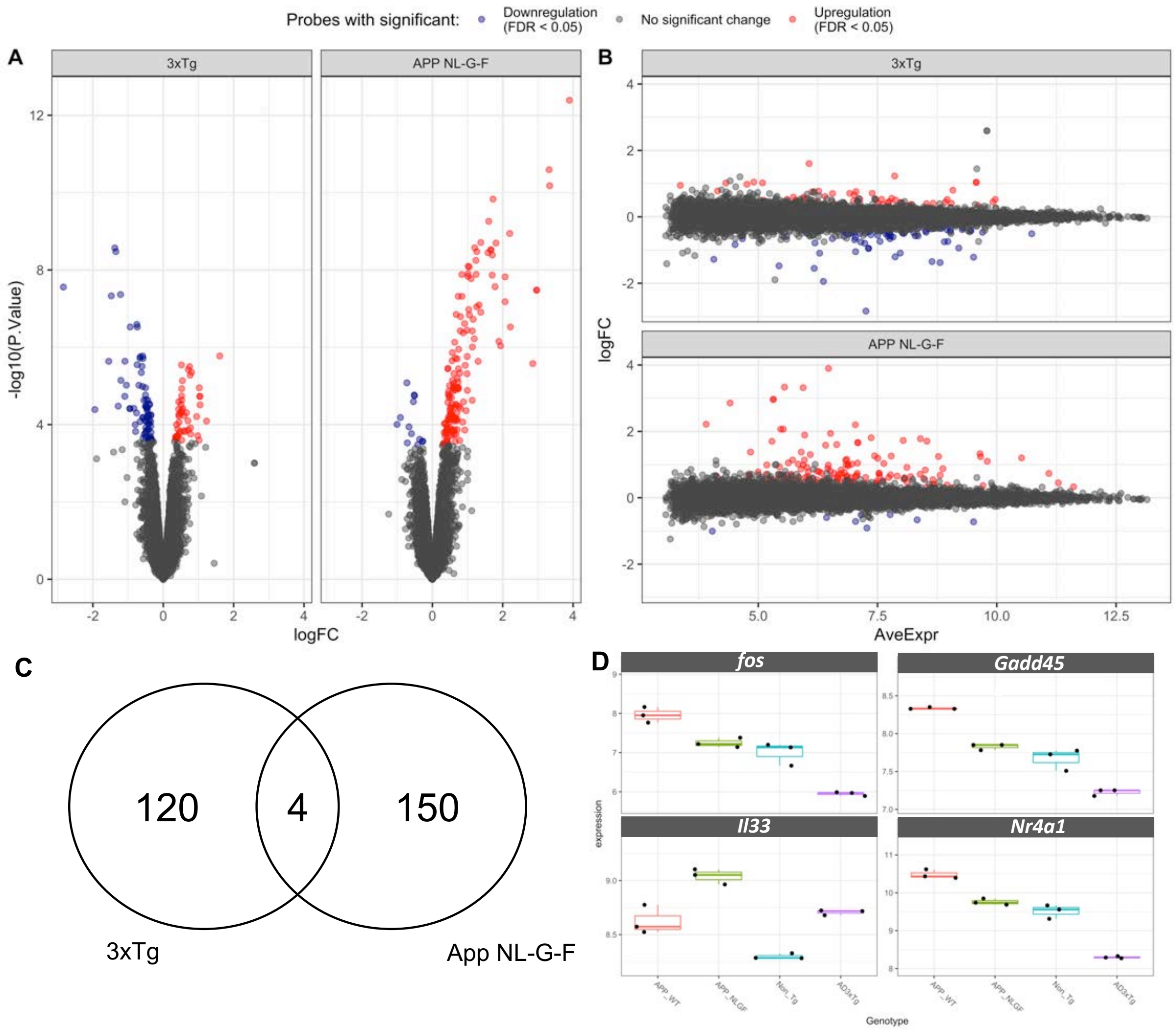

Fig. S8. Differential gene expression analysis of *APP* mutation mouse models. A) Volcano plot and **B)** MD plot of the changes to gene expression in $App^{NL\text{-}G\text{-}F/NL\text{-}G\text{-}F}$ and 3xTg mice relative to controls. **C)** Venn diagram showing four genes are identified as differentially expressed (DE) in both comparisons. **D)** Boxplot of expression values of the four shared DE genes. The FDR-adjusted p-values as calculated from the differential gene expression analysis using *limma* are indicated.

Disease Models & Mechanisms • Supplementary information

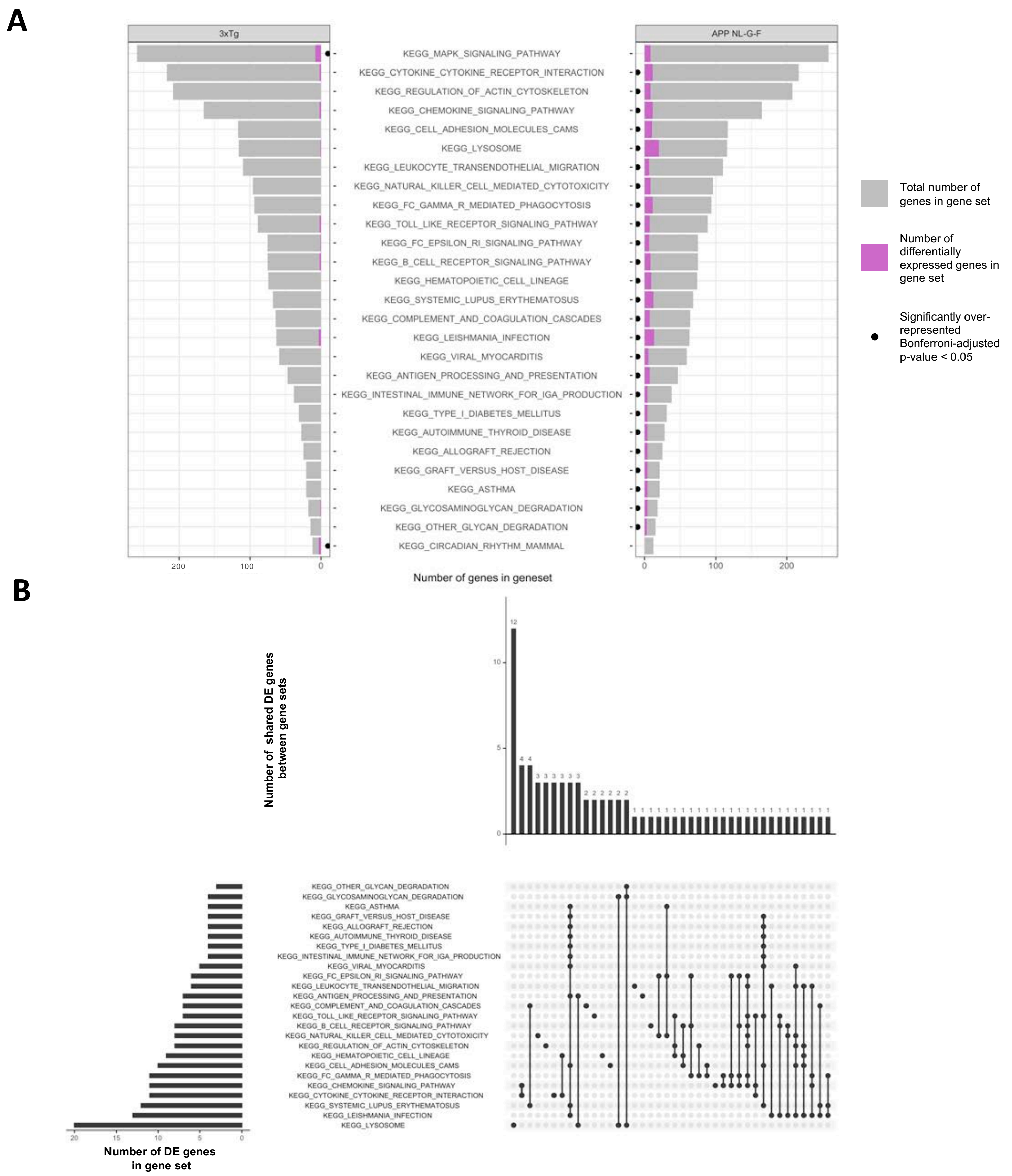

Disease Models & Mechanisms • Supplementary information

Fig. S9. Over-representation analysis of *APP* mutation mouse models. **A)** Pyramid bar plot indicating the number of genes in the significantly enriched KEGG and IRE gene sets in *App*$^{NL\text{-}G\text{-}F/NL\text{-}G\text{-}F}$ and 3xTg mice. Only gene sets with a Bonferroni adjusted p-value from *kegga* are shown (and are indicated by a black dot). The total numbers of genes in these gene sets are shown by grey bars, while the numbers of significantly differentially expressed (DE) genes in these gene sets are shown in magenta. **B)** Upset plot indicating the high degree of overlap of DE genes across the significantly enriched gene sets in *App*$^{NL\text{-}G\text{-}F/NL\text{-}G\text{-}F}$ mice.

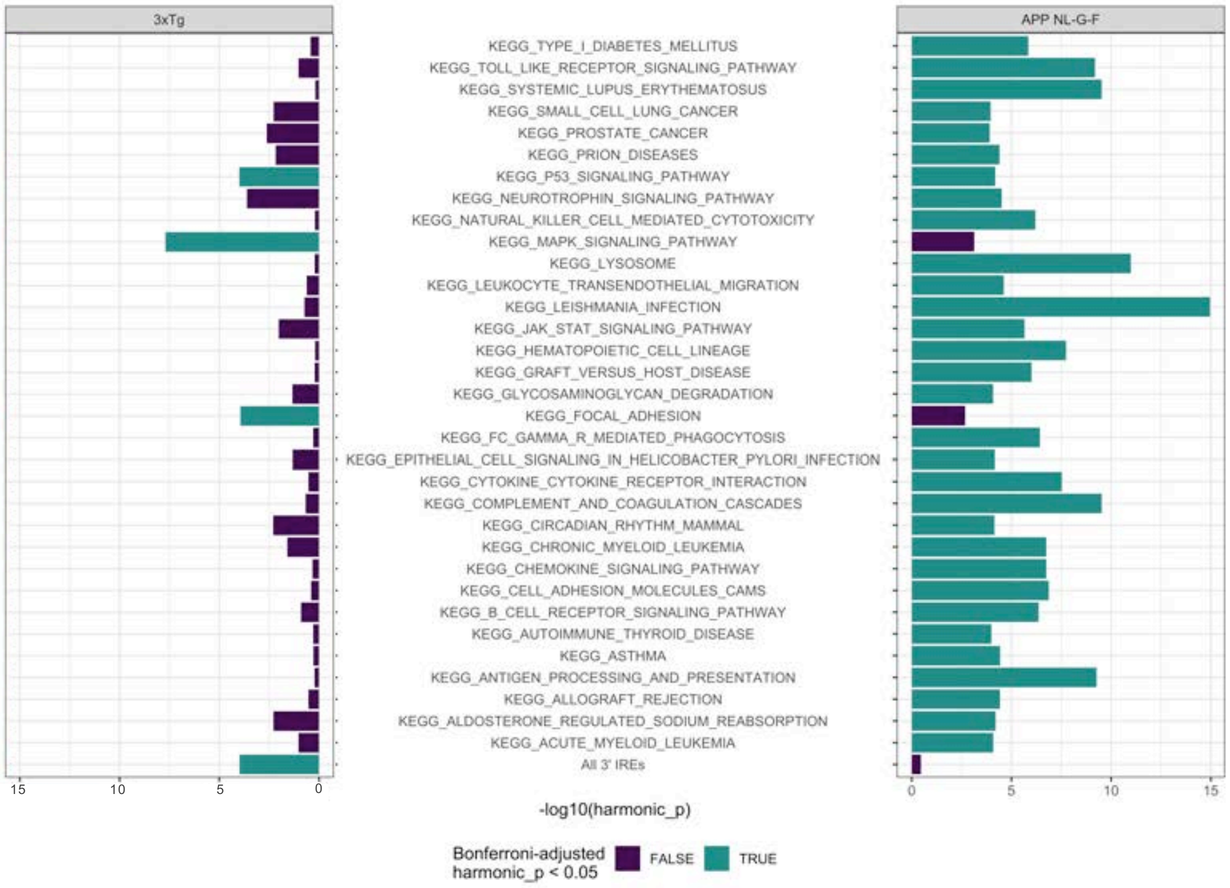

Fig. S10. Ranked-list enrichment testing. Summary of significantly enriched *KEGG* and *IRE* gene sets in *App*$^{NL\text{-}G\text{-}F/NL\text{-}G\text{-}F}$ and 3xTg mice. Gene sets are coloured according to whether they were below the threshold of a Bonferroni-adjusted harmonic mean p-value of < 0.05

Disease Models & Mechanisms • Supplementary information

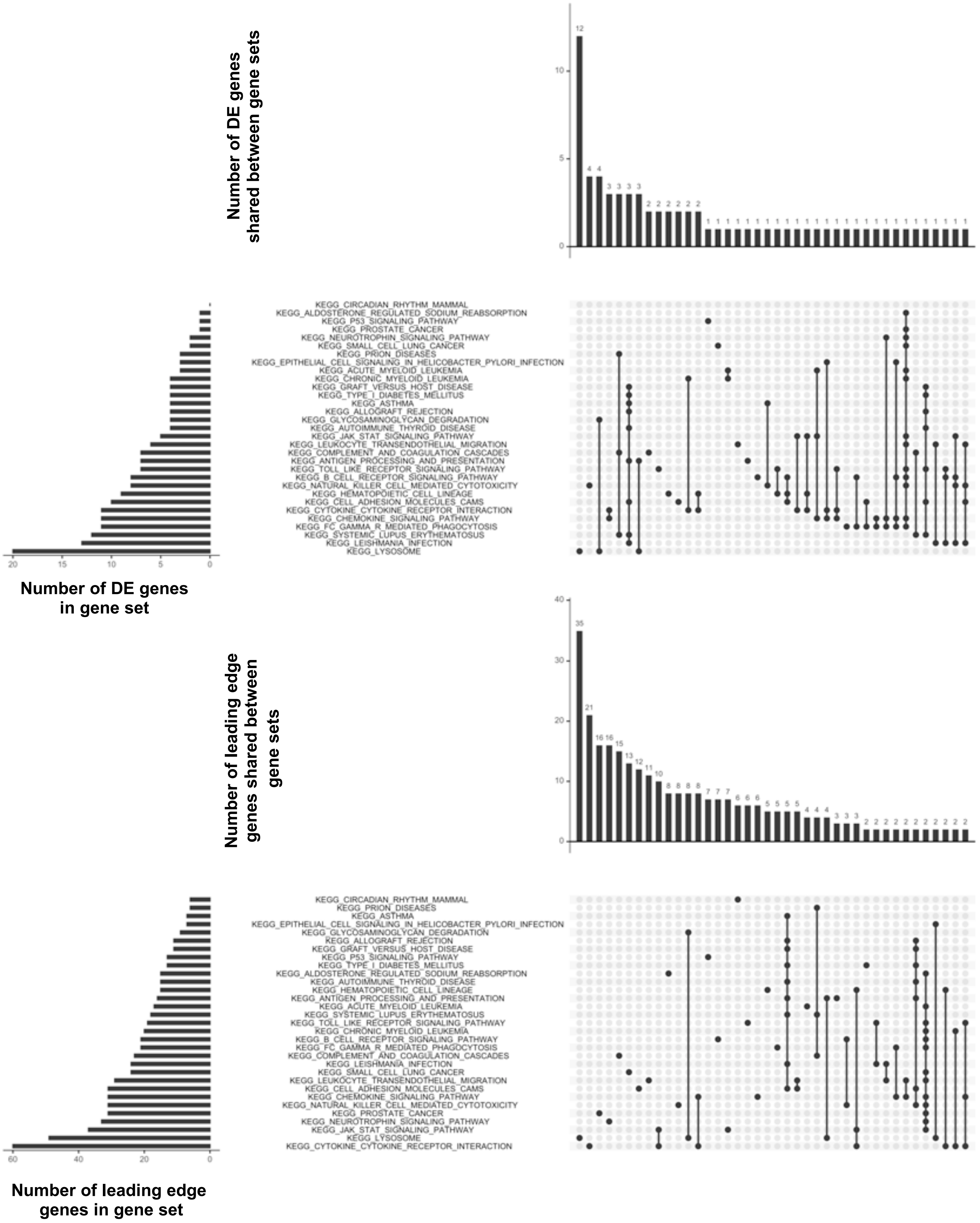

Fig. S11. Upset plots indicating the overlap of differentially expressed (DE, upper) and leading edge (lower) genes across the significantly altered gene sets in *App*NL-G-F/NL-G-F mice. DE genes were identified using *limma,* and the leading edge genes were obtained from the *GSEA* algorithm.

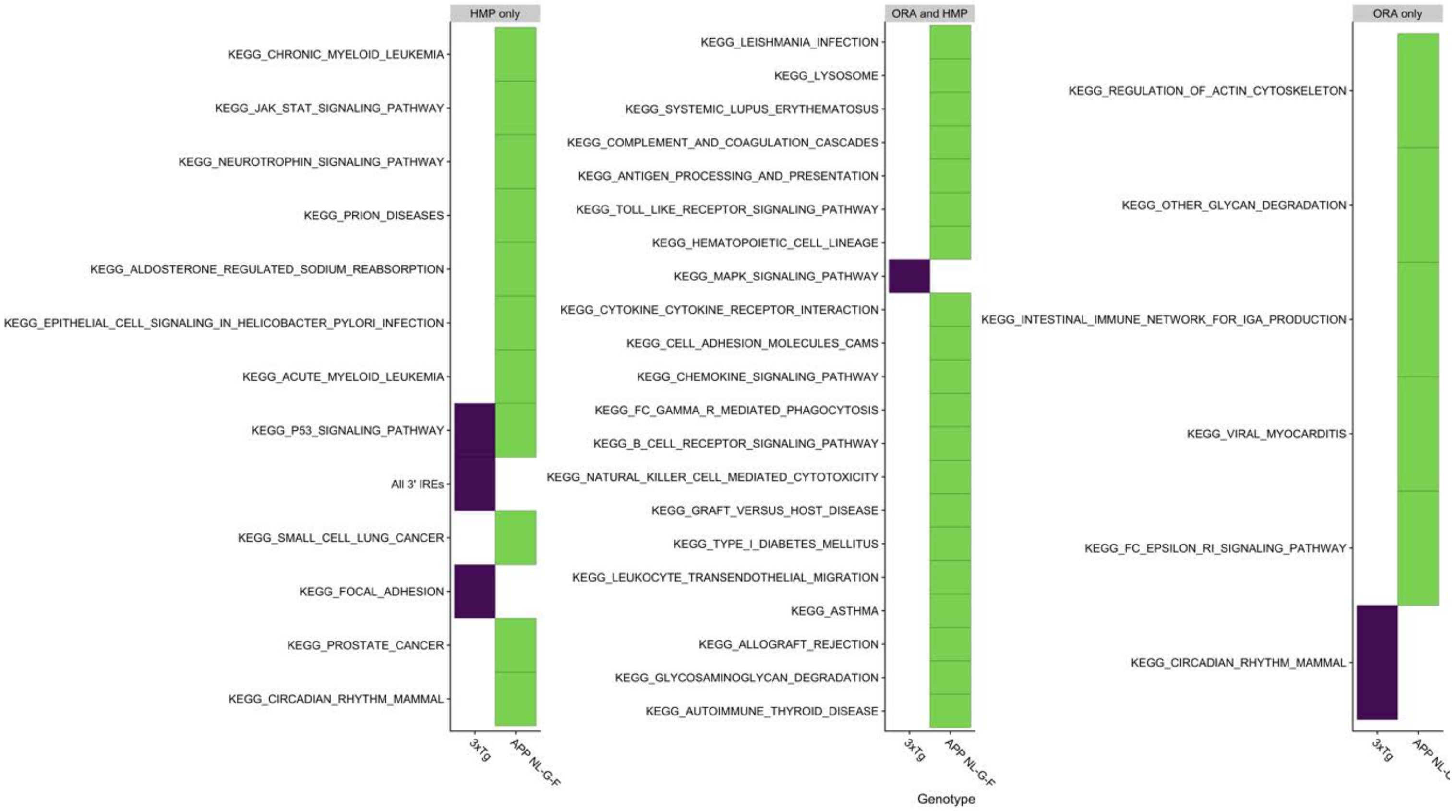

Fig. S12. Summary of significantly altered gene sets in *App*$^{NL\text{-}G\text{-}F/NL\text{-}G\text{-}F}$ and 3xTg mice. Gene sets only found to be altered by calculation of the harmonic mean p-value (HMP) are shown on the left. Gene sets only found to be altered by over-representation analysis (ORA) using *kegga* are shown on the right. Gene sets found to be altered in both types of enrichment are shown in the middle. Gene sets are coloured according to the comparison in which they are significantly altered.

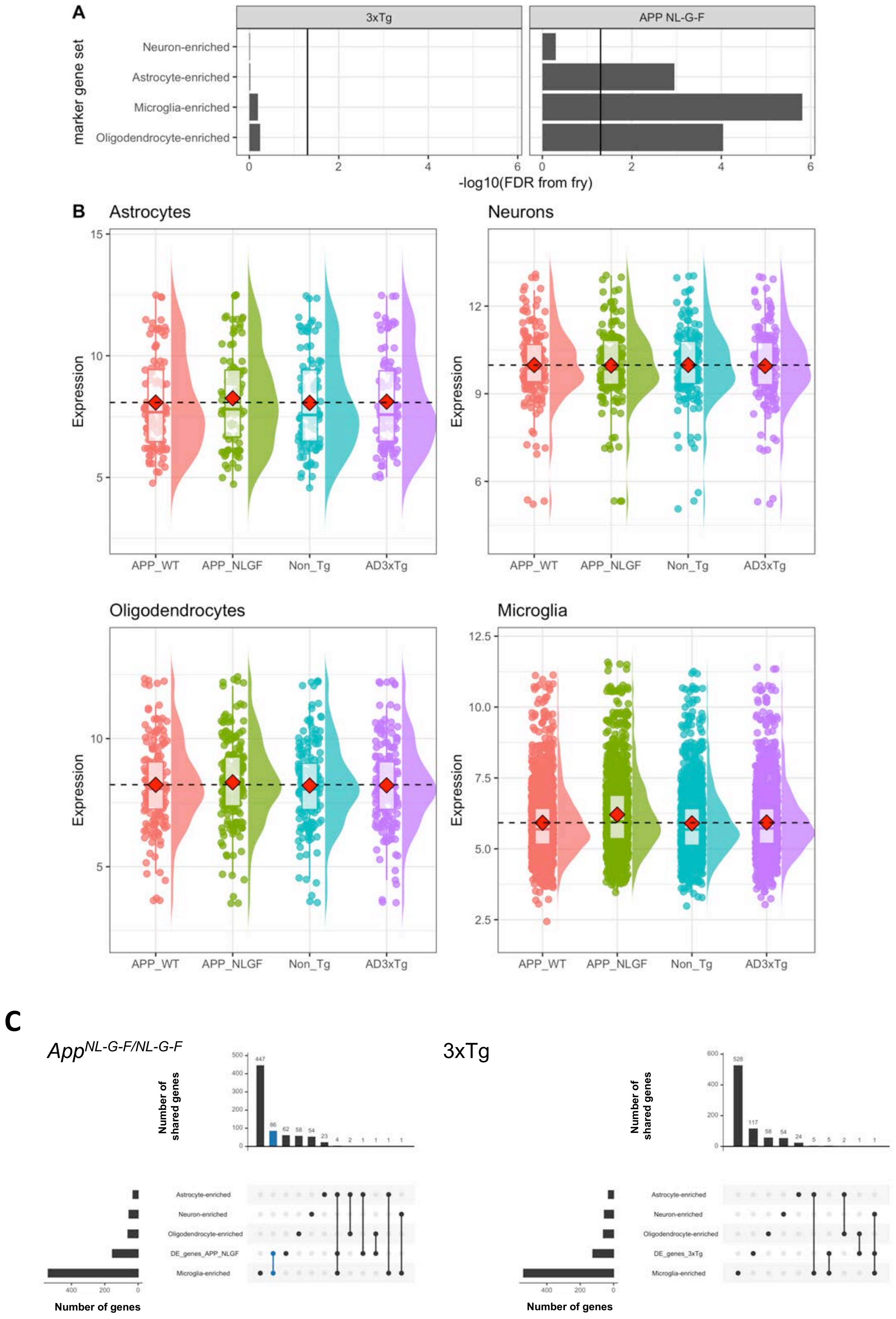

Disease Models & Mechanisms • Supplementary information

Fig. S13. Proportions of cell types in *App*$^{NL\text{-}G\text{-}F/NL\text{-}G\text{-}F}$ mice are altered. A) Gene set testing using *fry* on marker gene sets of neurons, astrocytes, oligodendrocytes and microglia from. The black line indicates an FDR-adjusted p-value of 0.05. **B)** Distribution of intensities of the marker genes across genotypes. The boxplots indicate summary statistics. The mean intensity value for each genotype is indicated by the red diamonds. To assist with visualisation of the increased expression of marker genes in *App*$^{NL\text{-}G\text{-}F/NL\text{-}G\text{-}F}$ mice, the mean expression in *App*$^{+/+}$ mice is also shown as a black dashed line. **C)** Upset plot showing the overlap of DE genes found in *App*$^{NL\text{-}G\text{-}F/NL\text{-}G\text{-}F}$ (left) and 3xTg (right) mice with the cell type marker gene sets.

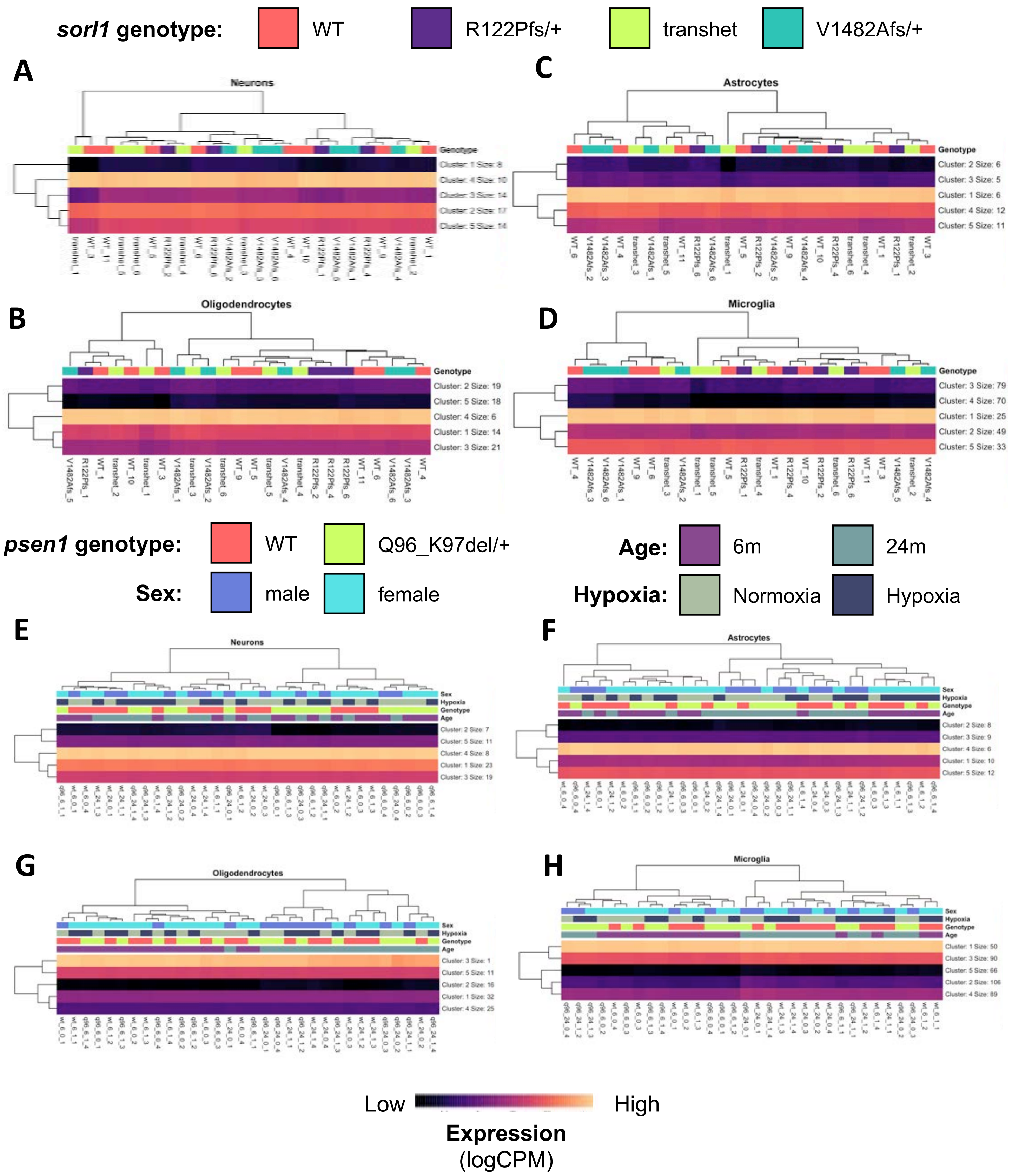

Fig. S14: Changes to cell type proportions are not observed in the brains of young, knock-in zebrafish models of EOfAD mutations. Expression values (log2 counts per million, logCPM) of marker genes of **A)** neurons (63 genes), **B)** astrocytes (40 genes), **C)** oligodendrocytes (78 genes) and **D)** microglia (256 genes) in *sorl1* knock-in mutant zebrafish. LogCPM values are also shown for *psen1* Q96_K97del mutant zebrafish in **E-H**. Rows represent clusters of genes with similar gene expression values summarised with k-means (k = 5). Columns represent samples, and are labelled with metadata of each experiment (see legends). Rows and columns are clustered based on their Euclidean distance. Samples do not cluster by genotype (or by genotype within age or treatment groups) in either experiment, meaning that expression of cell-type specific marker genes are consistent across genotypes.

Disease Models & Mechanisms • Supplementary information

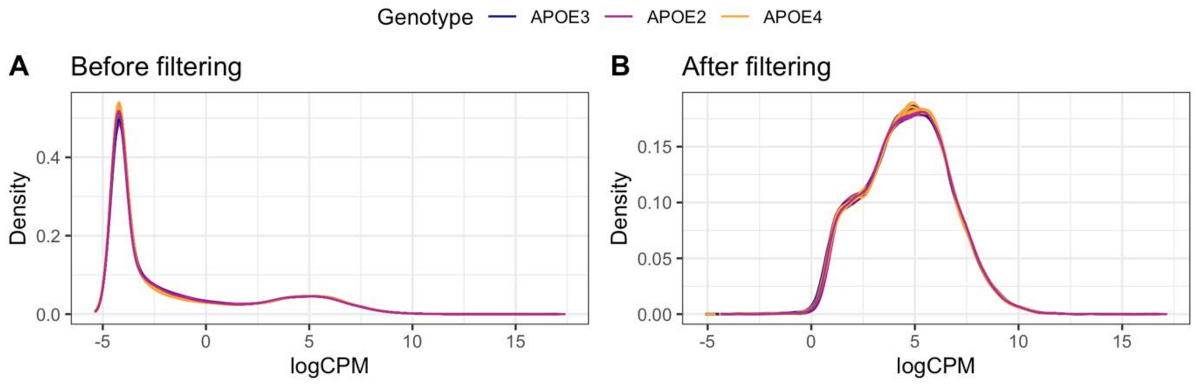

Fig. S15. The density of the log2 counts per million (logCPM) values detected in 3 month old APOE-TRmouse brain samples is shown before filtering in **A)**, then after omitting samples with a logCPM of < 2 in at least one third of the samples in **B)**.

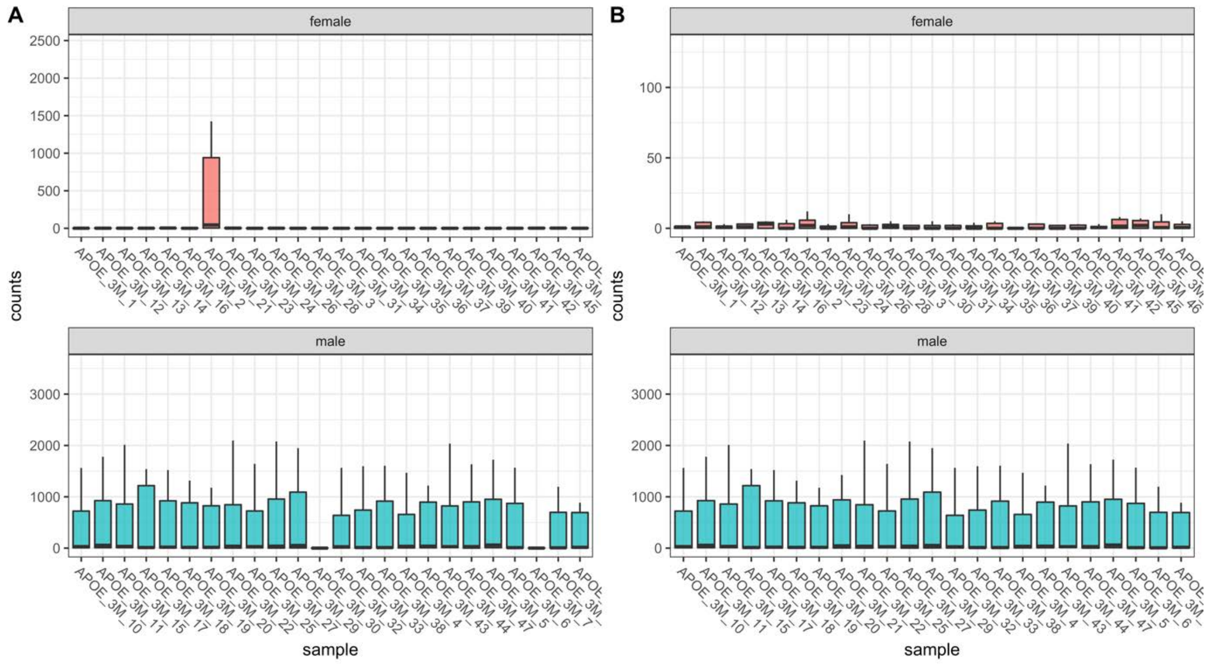

Fig. S16. Assessment of expression of genes from the Y-chromosome. A) Boxplots showing the summary statistics of the number of reads aligning to male-specific genes (located on the Y-chromosome) in the cortex of 3 month old APOE-TR mice, grouped by the metadata obtained from the AD Knowledge Portal database. Sample APOE_3M_21 appears to be a male sample as it expresses genes from the Y chromosome. Samples APOE_3M_30 and APOE_3M_7 appear to be female as they do not express genes from the Y-chromosome. **B)** Number of reads aligning to male-specific genes after correcting the sex of the samples.

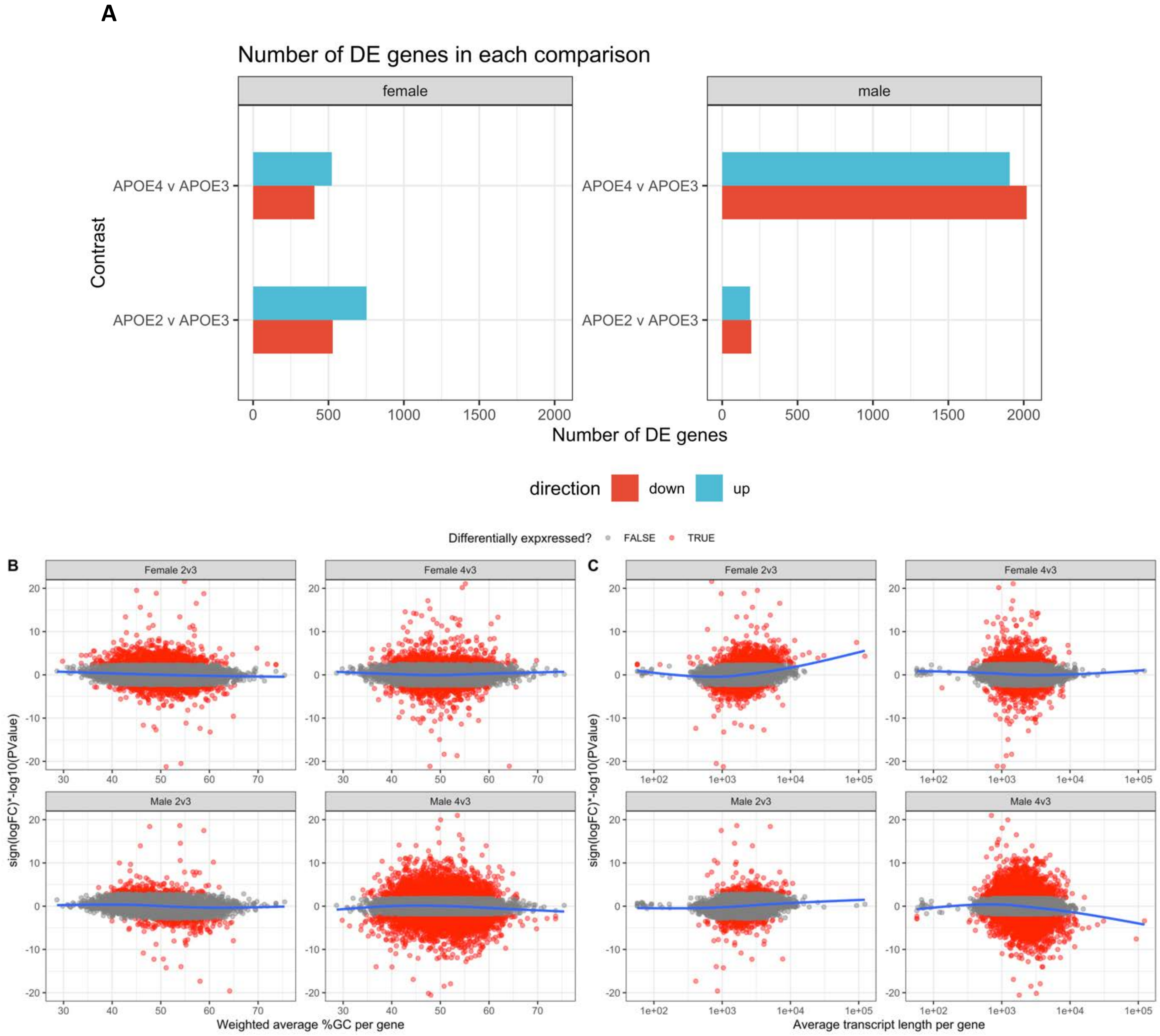

Fig. S17. 4: Initial differential expression analysis. A) Bar chart showing the number of differentially expressed genes (DE) in each comparison of the APOE4 or APOE2 genotype to APOE3. **B)** A ranking statistic per gene was calculated as the sign of the logFC multiplied by the negative log10 of the p-value from the likelihood ratio tests in *edgeR*. This was plotted against a weighted (by transcript length) average %GC content per gene and **C)** average transcript length. The blue generalised additive model fit (gam) lines are not centred on 0, indicating a bias.

Disease Models & Mechanisms • Supplementary information

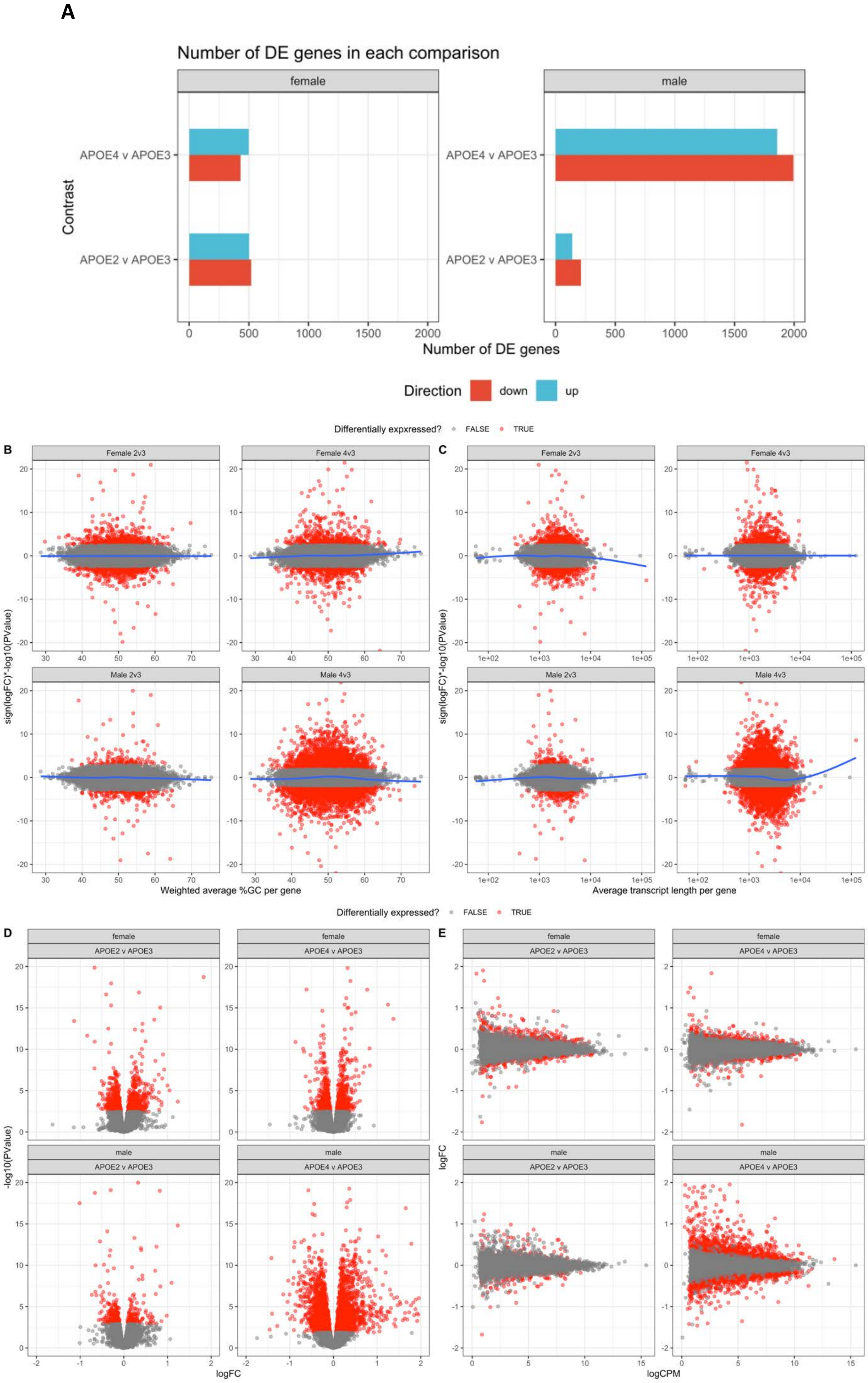

Disease Models & Mechanisms • Supplementary information

Fig. S18. Differential gene expression analysis after *cqn*. A) Number of genes identified to be differentially expressed (DE) after *cqn*. **B)** Improvement of observed bias between %GC content and **C)** gene length for differential expression after *cqn*. The remaining bias for transcript length in the female APOE2 and male APOE4 comparisons appear to be only driven by a small number of genes and can be ignored. **D)** Volcano plots and **E)** mean difference (MD) plots of the changes to gene expression observed due to homozygosity for the APOE4 or APOE2 alleles relative to APOE3 in male and female mice. The limits of the x-axis in **D)** and the y-axis in **E)** are constrained to -2 and 2, and of the y-axis in **D)** to between 0 and 20, for visualisation purposes.

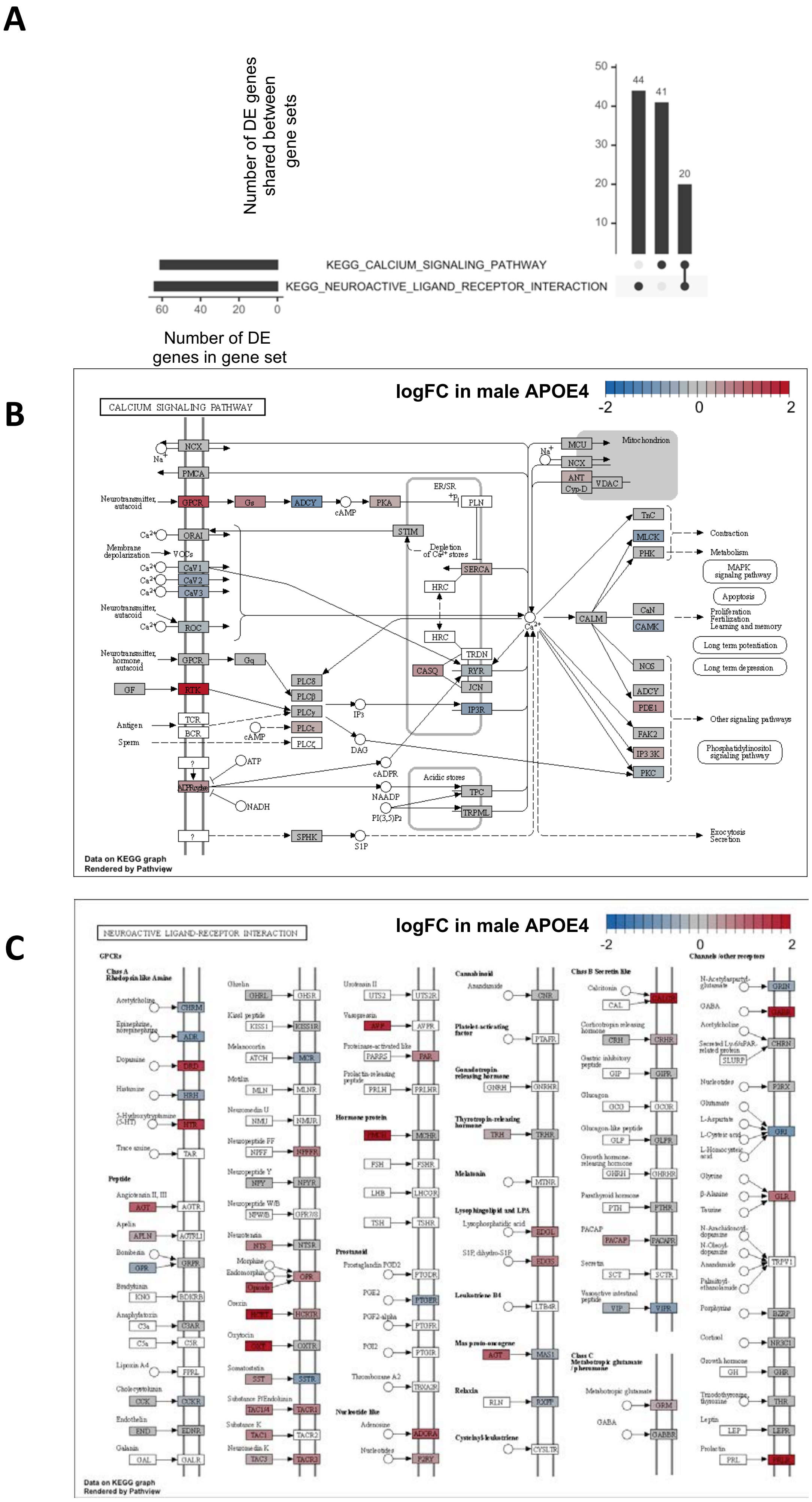

Fig. S19. Enrichment analysis within the lists of differentially expressed (DE) genes in APOE4 mice. **A)** Upset plot indicating the overlap of DE genes in male APOE4 samples for the two significantly enriched gene sets. **B)** Pathview visualisation of the logFC in male APOE4 samples for the *KEGG_CALCIUM_SIGNALING_PATHWAY* gene set and **C)** *KEGG_NEUROACTIVE_LIGAND_RECEPTOR_INTERACTION* gene set.

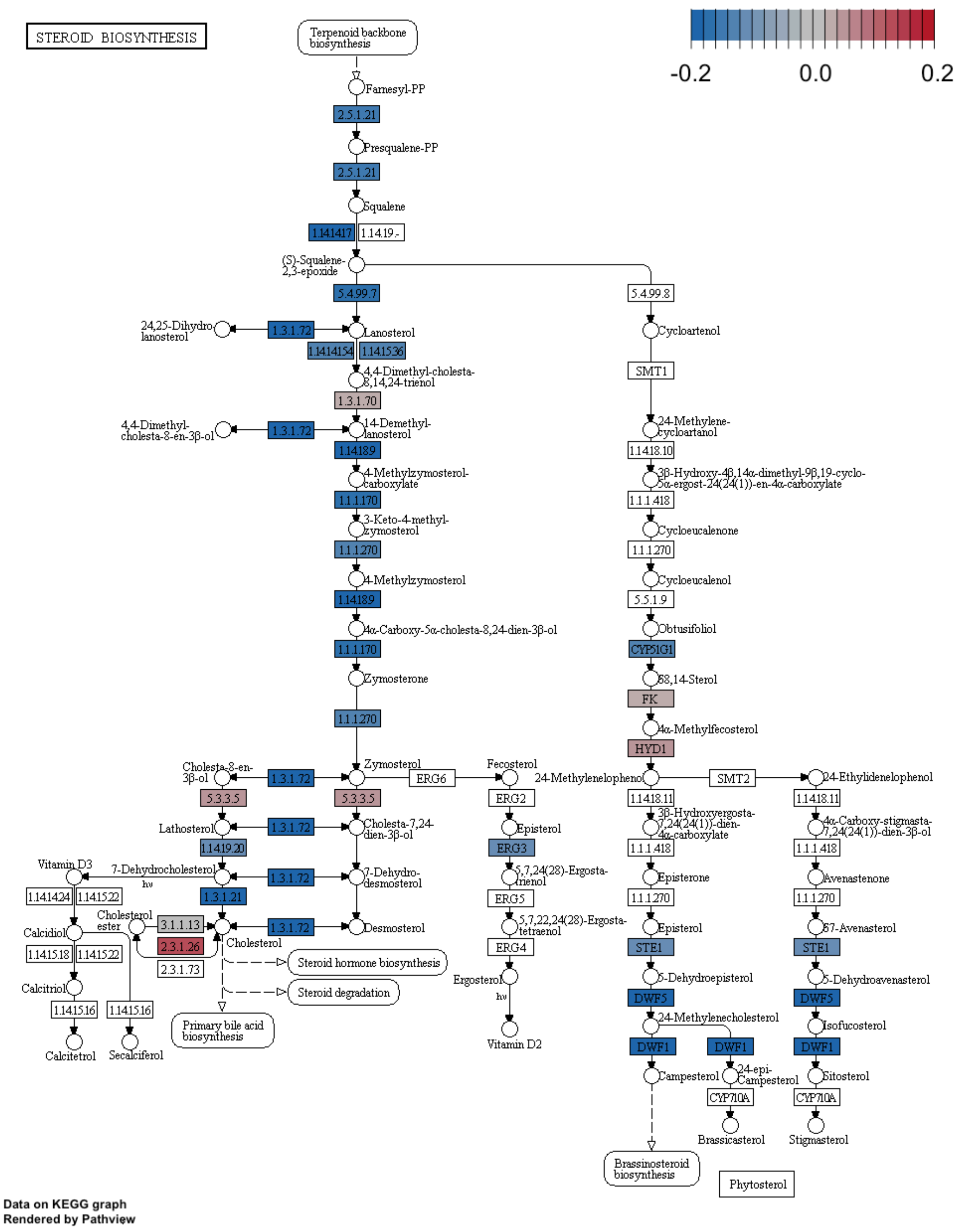

Fig. S20. Pathview visualisation indicating the logFC of genes in the *KEGG_STEROID_BIOSYNTHESIS* gene set in female APOE2 mice.

Disease Models & Mechanisms • Supplementary information

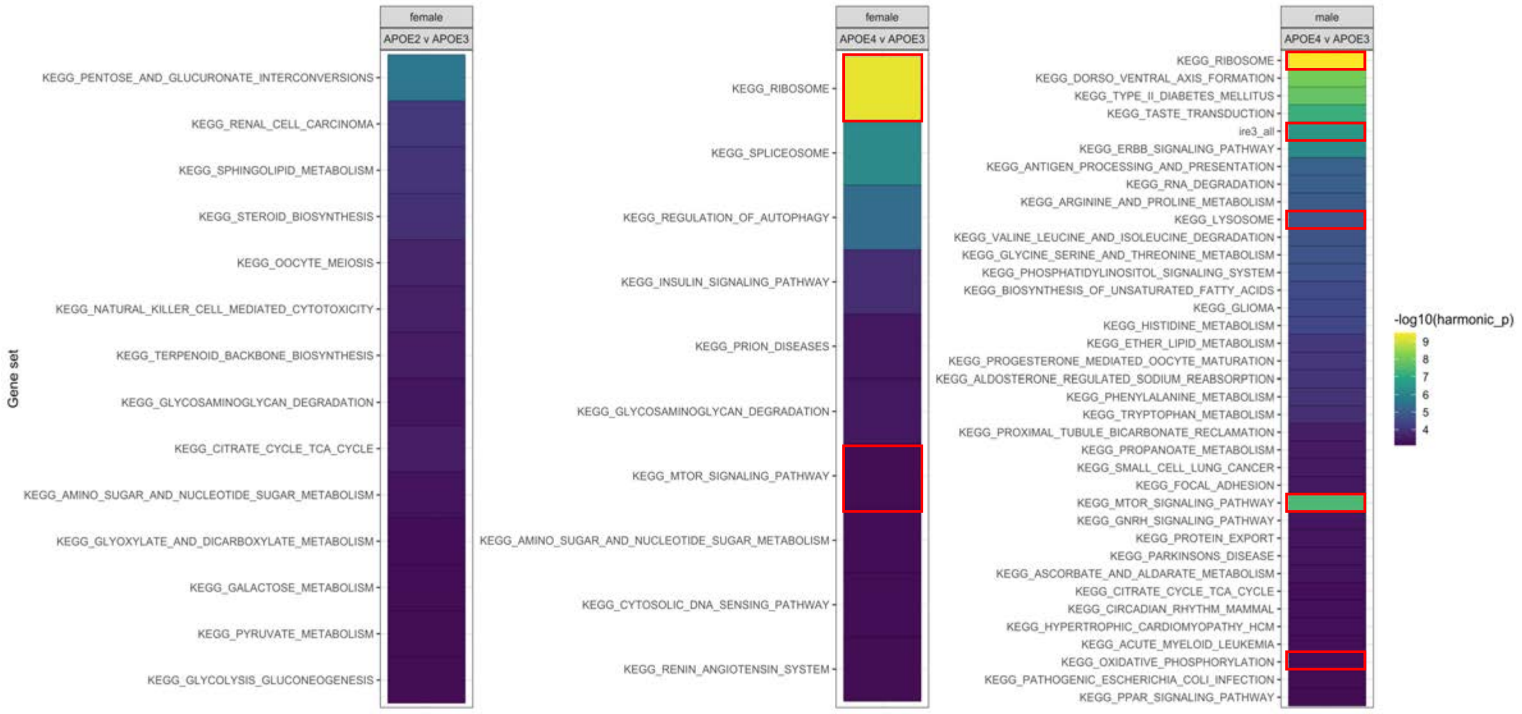

Fig. S21. Heatmap indicating gene sets with a FDR-adjusted harmonic mean p-value < 0.01 in APOE-TR mice at 3 months of age. Gene sets of interest are highlighted with a red box. Note that no gene sets were found to contain an FDR-adjusted harmonic mean p-value of < 0.01 in male APOE2 mice

Disease Models & Mechanisms • Supplementary information

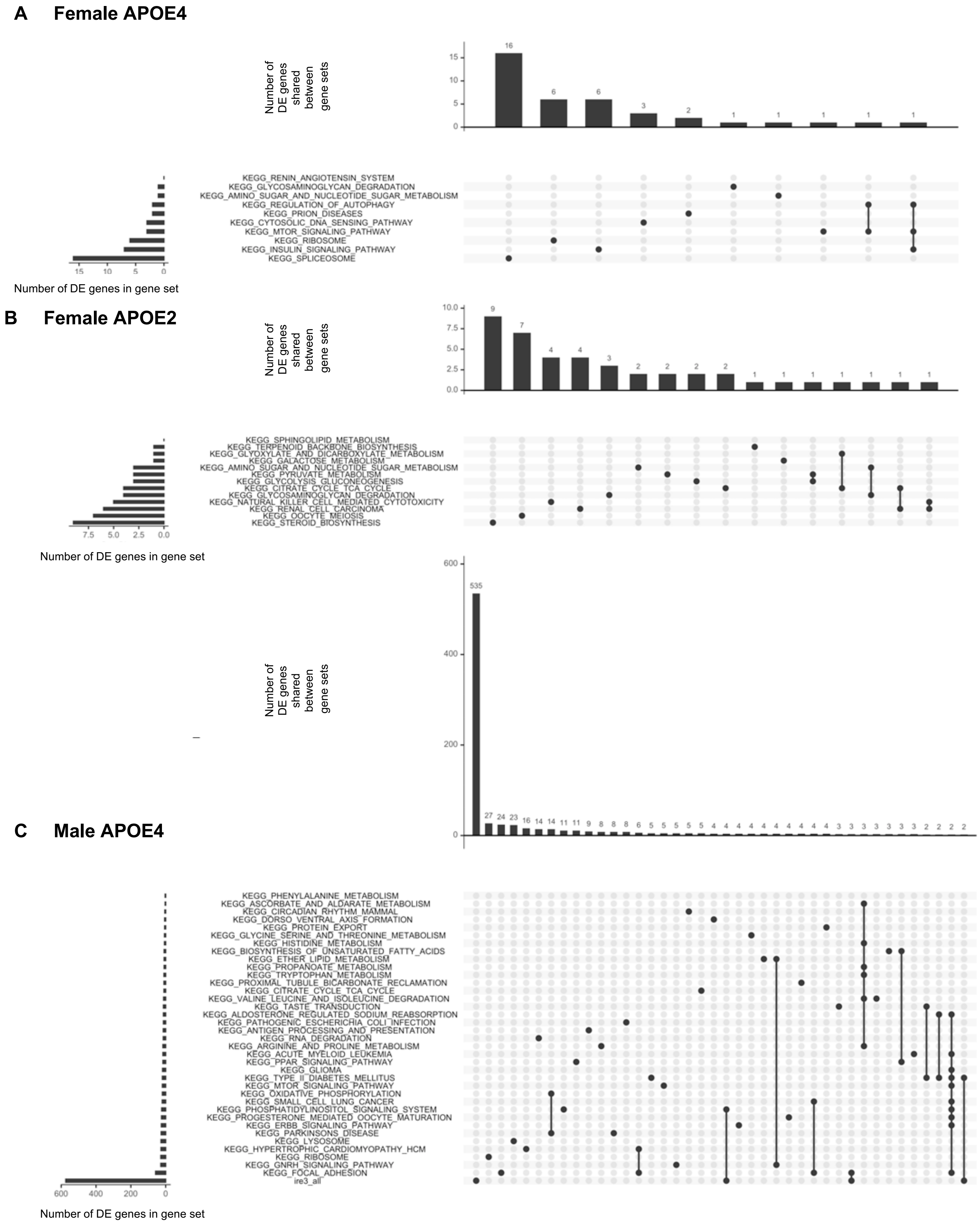

Fig. S22. Upset plots indicating the overlap of DE genes across the gene sets which were calculated to have a FDR-adjusted harmonic mean p-value < 0.01 in **A)** female APOE4 mice, **B)** female APOE2 mice and **C)** male APOE4 mice. Note that the *KEGG* gene sets for oxidative phosphorylation and Parkinson's disease share 14 DE genes.

Disease Models & Mechanisms • Supplementary information

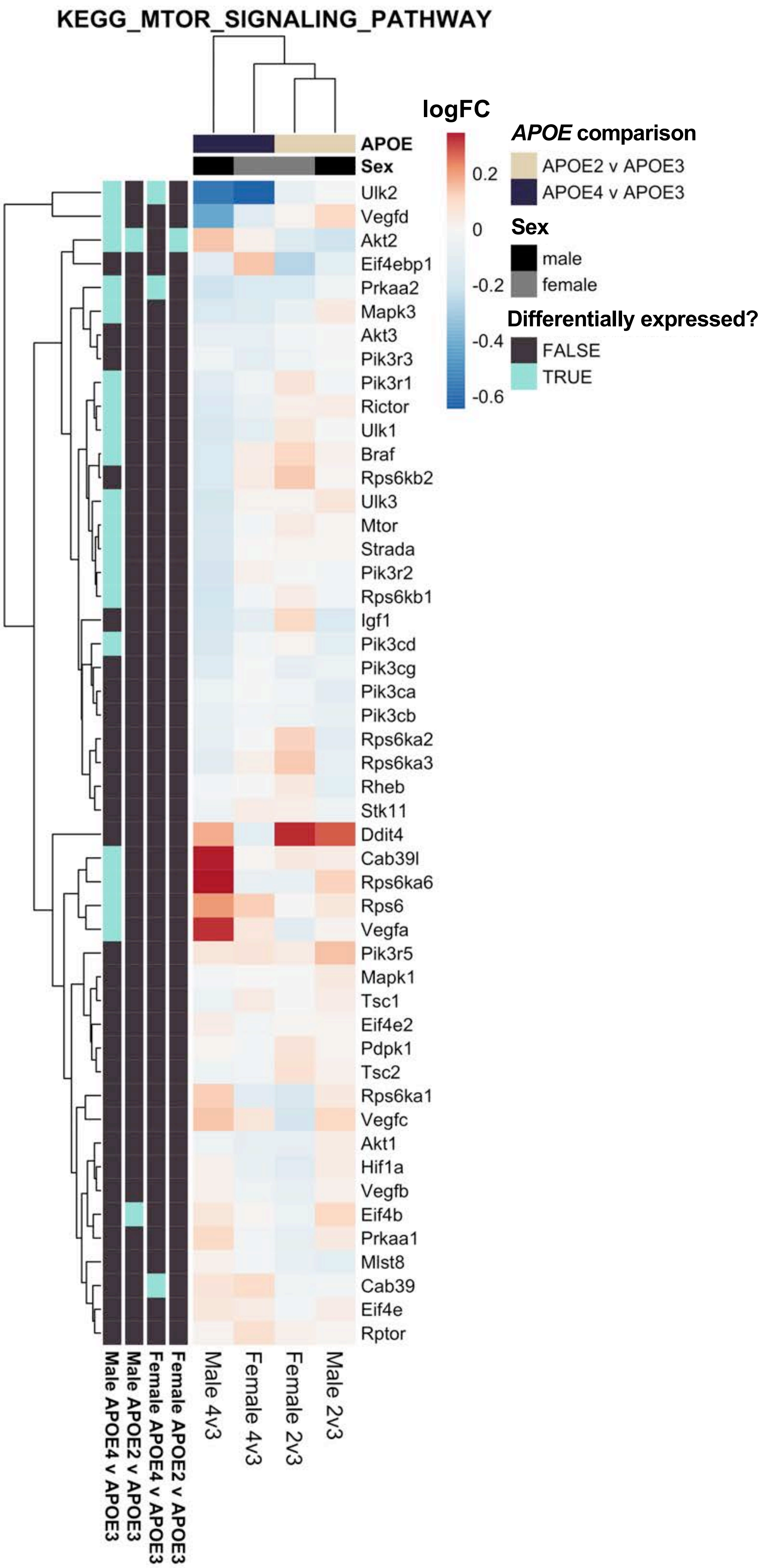

Fig. S23. Heatmap showing the log2 fold change (logFC) of genes detected in APOE-TR mice in the *KEGG_MTOR_SIGNALING_PATHWAY* gene set. Rows (genes) and columns (comparisons) are clustered based on their Euclidean distance. Genes are labelled whether they were found to be significantly differentially expressed (DE) in the DE analysis with *edgeR*. Columns are labelled with the *APOE* genotype and sex comparison.

Disease Models & Mechanisms • Supplementary information

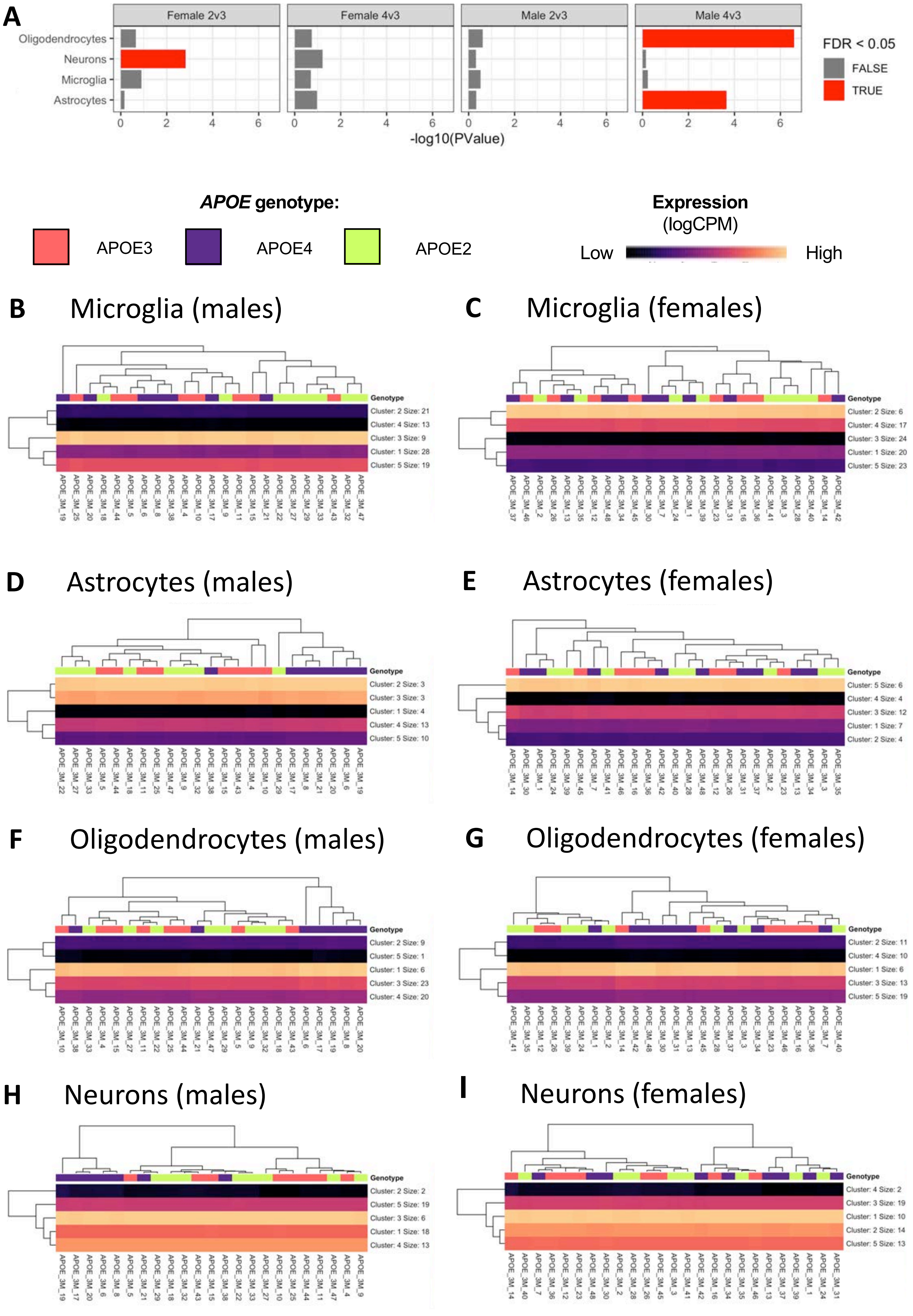

Fig. S24. Changes to cell type proportions in APOE-TR mice. A) Significance of gene set testing from fry with a directional hypothesis of gene sets consisting of marker genes of neurons, astrocytes, oligodendrocytes and microglia. **B-I)** Expression (logCPM) of these cell type marker genes in APOE-TR mice. Rows represent clusters of genes with similar gene expression values summarised with k-means (k = 5). Columns represent samples, and are labelled with *APOE* genotype (see legend). Rows and columns are clustered based on their Euclidean distance. Male APOE4 samples mostly form distinct clusters in **D)** and **F)**, indicating that expression of cell-type specific marker genes are distinct in these genotypes.

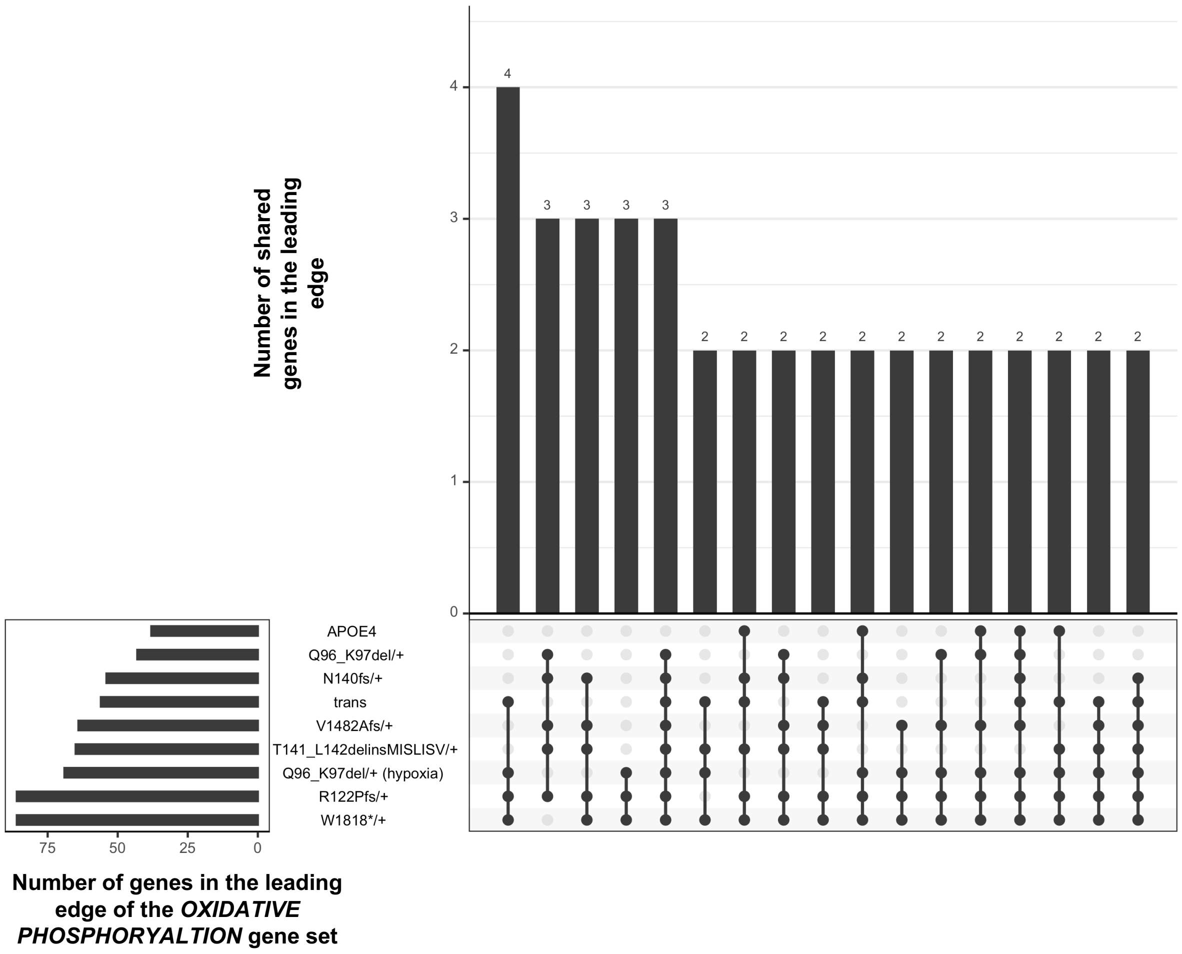

Fig. S25. The genes which drive the statistical significance of the *KEGG_OXIDATIVE_PHOSPHORYALTION* gene set are frequently different in male APOE4 mice and EOfAD model zebrafish. The upset plot shows the overlap of genes in the leading edge of the *KEGG_OXIDATIVE_PHOSPHORYALTION* gene set from the *fgsea* algorithm. Only genes which were found in the leading edge for at least two mutations are displayed.

Disease Models & Mechanisms • Supplementary information

Table S1. Gene sets significantly enriched with differentially expressed genes in APOE-TR mice.

Gene set	FDR	Coef
KEGG_CALCIUM_SIGNALING_PATHWAY	0.03	APOE4 male
KEGG_NEUROACTIVE_LIGAND_RECEPTOR_INTERACTION	0.04	APOE4 male
KEGG_STEROID_BIOSYNTHESIS	1e-5	APOE2 female

Table S2. Significance of the *KEGG_OXIDATIVE_PHOSPHORYLATION* gene set in young APOE-TR mice.

Sex	*APOE*	FDR-adjusted harmonic mean p-value
Male	APOE4	0.00948
	APOE2	0.794
Female	APOE4	0.794
	APOE2	0.248

Table S3: Significance of the *KEGG_RIBOSOME* gene set in young APOE-TR mice.

Sex	*APOE*	FDR-adjusted harmonic mean p-value
Male	APOE4	0.0000000782
	APOE2	0.691
Female	APOE4	0.000000101
	APOE2	0.793
